# An efficient consolidation of word embedding and deep learning techniques for classifying anticancer peptides: FastText+BiLSTM

**DOI:** 10.7717/peerj-cs.1831

**Published:** 2024-02-20

**Authors:** Onur Karakaya, Zeynep Hilal Kilimci

**Affiliations:** 1 Research and Development Inc., Turkcell Technology, İstanbul, Turkey; 2 Department of Information Systems Engineering, Kocaeli University, Kocaeli, Turkey

**Keywords:** Anticancer peptides, Word embeddings, Deep learning, FastText, Word2Vec, CNN, LSTM, BiLSTM

## Abstract

Anticancer peptides (ACPs) are a group of peptides that exhibit antineoplastic properties. The utilization of ACPs in cancer prevention can present a viable substitute for conventional cancer therapeutics, as they possess a higher degree of selectivity and safety. Recent scientific advancements generate an interest in peptide-based therapies which offer the advantage of efficiently treating intended cells without negatively impacting normal cells. However, as the number of peptide sequences continues to increase rapidly, developing a reliable and precise prediction model becomes a challenging task. In this work, our motivation is to advance an efficient model for categorizing anticancer peptides employing the consolidation of word embedding and deep learning models. First, Word2Vec, GloVe, FastText, One-Hot-Encoding approaches are evaluated as embedding techniques for the purpose of extracting peptide sequences. Then, the output of embedding models are fed into deep learning approaches CNN, LSTM, BiLSTM. To demonstrate the contribution of proposed framework, extensive experiments are carried on widely-used datasets in the literature, ACPs250 and independent. Experiment results show the usage of proposed model enhances classification accuracy when compared to the state-of-the-art studies. The proposed combination, FastText+BiLSTM, exhibits 92.50% of accuracy for ACPs250 dataset, and 96.15% of accuracy for the Independent dataset, thence determining new state-of-the-art.

## Introduction

Cancer, a widespread and devastating illness marked by uncontrolled cell growth and metastasis, remains a prominent cause of global mortality. The World Health Organization reports that various cancer types lead to millions of annual deaths, with this number steadily increasing. Recent data indicates that in 2020, the global count of newly diagnosed cancer cases exceeded 19.3 million, resulting in around 10 million fatalities (Matthews, Bertoli & de Bruin, 2022). The COVID-19 pandemic significantly disrupted cancer diagnosis and treatment due to healthcare facility closures, job losses, health insurance disruptions, and concerns about COVID-19 exposure. Although the most substantial impact was observed in mid-2020, the healthcare system has not fully recovered. Notably, Massachusetts General Hospital saw a decline in surgical oncology procedures, reaching only 72% of 2019 levels in the latter half of 2020 and modestly recovering to 84% in 2021, the lowest rebound among surgical specialties (Ghoshal et al., 2022). Delays in cancer diagnosis and treatment introduction can contribute to increased advanced-stage disease prevalence and subsequent mortality rates (Yabroff et al., 2022).

Traditional cancer treatments, such as chemotherapy and radiation therapy, often have limitations in effectiveness, selectivity, and adverse side effects. While conventional anticancer therapies, including chemotherapy and radiation, exhibit high efficacy, they come with substantial costs and undesired side effects on healthy cells. Additionally, cancer cells can develop resistance mechanisms against conventional chemotherapeutic agents (Holohan et al., 2013). Treatments like radiation therapy, targeted therapy, and chemotherapy frequently yield suboptimal results and cause numerous side effects in recipients, including cognitive impairment, insomnia, gastrointestinal problems, compromised immune systems, thrombocytopenia, and anemia (Thundimadathil, 2012; Maliepaard et al., 2001). Hence, there is an urgent need for more effective treatments to address these drawbacks.

In recent years, peptide-based therapy has emerged as a promising approach for cancer treatment. Anticancer peptides (ACPs) have shown promise due to their ability to selectively target and disrupt cancer cell membranes, interfere with intracellular processes, and induce apoptosis. ACPs typically refer to short peptide fragments derived from protein sequences, containing fewer than 50 amino acids (Tyagi et al., 2015). Various peptide-based therapeutic approaches have been employed in clinical and preclinical trials for treating different tumor types (Gregorc et al., 2011; Khalili, 2006). The identification and classification of ACPs traditionally rely on experimental techniques such as high-throughput screening and mass spectrometry. However, these approaches are time-consuming, labor-intensive, and limited by the availability of peptide libraries. To overcome these challenges, computational methods have emerged as valuable tools for predicting and classifying ACPs (Boopathi et al., 2019).

Indeed, nowadays, deep learning (DL) technology has become a focal point in the fields of machine learning (ML), artificial intelligence (AI), data science, and analytics. This prominence is due to its exceptional ability to extract insights from provided data. Within its operational domain, DL is recognized as a subset of both ML and AI, representing an AI function that emulates the cognitive data processing capabilities of the human brain. Notably, DL distinguishes itself from traditional ML by demonstrating increased efficiency as data volumes grow. Through the use of multiple layers to capture data abstractions, DL constructs computational models that facilitate intricate learning processes. Although DL requires extensive training time due to a substantial number of parameters, its testing phase exhibits noteworthy efficiency compared to other ML algorithms, necessitating less computational time (Xin et al., 2018). In recent years, ML has found application in various domains such as encompassing text mining, spam detection, video recommendation, image classification, and multimedia concept retrieval. In Rozenwald et al. (2020), linear regression models with four types of regularization, gradient boosting, and recurrent neural networks (RNN) are presented as tools for investigating chromatin folding characteristics linked to TADs using epigenetic chromatin immunoprecipitation data. The study Amrit et al. (2017) involves the examination of whether meaningful patterns are identified in the consultation data to detect abuse using machine learning techniques namely, naive Bayes, support vector machine, and random forest. A comprehensive study Hossain et al. (2019) is conducted in this paper, focusing on the utilization of big data and machine learning within the electrical power grid context, as introduced by the next-generation power system known as the smart grid. In Crawford et al. (2015), a survey is carried out on the prominent machine learning techniques that have been suggested for addressing the issue of review spam detection, and the performance of various approaches in the classification and identification of review spam is assessed. In Al-Dulaimi et al. (2019), a method of linear discriminant analysis is proposed in which bispectral invariant features from a segmented Human Epithelial Type-2 (HEp-2) specimen cell shape are used to enable the generalization of features to variations in shape, rotation, scaling, and shifting.

DL is also recognized as representation learning (RL). The increasing enthusiasm in the realms of deep and distributed learning can be ascribed to the exponential surge in data availability and the noteworthy advancements in hardware technologies, such as High-Performance Computing (HPC) (Potok et al., 2018). DL extends the principles of conventional neural networks but notably outperforms its precursors in terms of performance. Moreover, DL integrates transformations and graph-based methodologies to construct complex, multi-layer learning models. DL, among the different ML algorithms, has emerged as a widely adopted technique in different applications. In Liu et al. (2017), an overview is presented in which some widely-used deep learning architectures, namely autoencoder, convolutional neural network, deep belief network, and restricted Boltzmann machine, and their practical applications are detailed. In Pouyanfar et al. (2018), a thorough review is presented, covering historical and current state-of-the-art approaches in visual, audio, and text processing, as well as social network analysis and natural language processing using deep learning architectures. In Alom et al. (2019), a concise overview is presented regarding the advances observed in the field of DL, commencing with the deep neural network (DNN). Subsequently, coverage is provided for various components, including the convolutional neural network (CNN), recurrent neural network (RNN) encompassing long short-term memory (LSTM) and gated recurrent units (GRU), auto-encoder (AE), deep belief network (DBN), generative adversarial network (GAN), and deep reinforcement learning (DRL).

Recent advancements in DL techniques have demonstrated exceptional performance across diverse applications, including audio and speech processing, visual data processing, and natural language processing (NLP). In Adeel, Gogate & Hussain (2020), a novel context-aware AV speech enhancement framework is introduced in which clean audio is estimated using a CNN and a LSTM network, without the necessity of prior SNR estimation. In the study Tian, Chen & Shyu (2020), a framework based on evolution programming is proposed to tackle various challenges using InceptionV3 and ResNet50 models. Young et al. (2018) reviews significant models and methods related to deep learning that have been utilized for various NLP tasks and offers an overview of their development. In Koppe, Meyer-Lindenberg & Durstewitz (2021), a comprehensive overview is provided how deep learning approaches can be utilized for prediction in psychiatry.

In the context of anticancer peptide classification, we hypothesize that the integration of advanced embedding techniques (Word2Vec, GloVe, FastText, and One-Hot-Encoding) with deep learning models (CNN, LSTM, BiLSTM) will significantly improve classification accuracy. Our hypothesis is rooted in the belief that combining semantic word embeddings with powerful sequence analysis will enhance the model’s ability to distinguish anticancer peptides, ultimately outperforming existing methods. To accomplish this objective, an effective model is presented for the classification of anticancer peptides, incorporating the fusion of word embedding techniques and deep learning models. The rationale behind the proposed approach is to assess and contrast the efficacy of cutting-edge deep learning models that strive to attain elevated accuracy in discerning between anticancer peptides and non-anticancer peptides. In order to illustrate the contribution of the proposed framework, two distinct benchmark datasets, namely ACPs250 and Independent, are utilized, ensuring a equitable comparison with contemporary studies in the field. Furthermore, the impact of different vector models, namely Word2Vec, GloVe, FastText, One-Hot-Encoding, is investigated to get robust classification results. Experiments results demonstrate that the inclusion of word embedding models when combined with deep learning architectures significantly improves the classification accuracy for detecting anticancer peptides.

The primary contributions of this article encompass the following aspects:

• **Evaluation of embedding techniques:** We systematically assess the performance of Word2Vec, GloVe, FastText, and One-Hot-Encoding as embedding techniques for extracting peptide sequences. Our contribution lies in providing a comprehensive comparison of these approaches to determine the most effective method for capturing semantic information in peptide data.

• **Integration of deep learning models:** We leverage deep learning models, including CNN, LSTM, and BiLSTM, to process the output of embedding techniques. Our contribution is the integration of these powerful models, which enable the capture of intricate sequence patterns, enhancing the overall classification process.

• **Efficient anticancer peptide classification:** By combining embedding models with deep learning techniques, we propose an efficient and accurate model for anticancer peptide classification. The outcome is a reliable and precise predictive tool for distinguishing anticancer peptides, which is especially valuable in cancer prevention and therapeutic applications.

• **State-of-the-art results:** Our experimental results demonstrate that the proposed framework, particularly the combination of FastText+BiLSTM, achieves state-of-the-art classification accuracy. This advancement contributes to the field of anticancer peptide research by offering a new benchmark for accurate predictions, furthering the development of peptide-based therapies.

• **Datasets:** We conduct extensive experiments on widely-used datasets, ACPs250 and Independent, to validate the effectiveness of our model. Our contribution includes the rigorous evaluation and validation of the proposed framework on real-world datasets, ensuring its practical applicability.

• **Safety and selectivity:** Our work contributes to the field of cancer therapeutics by emphasizing the potential of anticancer peptides in offering a higher degree of safety and selectivity, thus providing a viable alternative to conventional cancer treatments.

The rest of this article is designated as follows: Section “Related Work” gives a summary of related work on the classification of anticancer peptides. Section “Models” introduces the models used in this work. Section “Proposed Framework” describes the proposed framework. Section “Experiment Results” and Section “Conclusions” present experiment results, and conclusions, respectively. Portions of this text were previously published (https://arxiv.org/ftp/arxiv/papers/2309/2309.12058.pdf) as part of a preprint.

## Related Work

In this section, a brief literature studies on anti-cancer peptide classification is presented.

In Aziz et al. (2022), a novel multi-channel CNN is proposed for the identification of anticancer peptides (ACPs) from protein sequences. The data from state-of-the-art methodologies is collected and subjected to binary encoding for data preprocessing. Additionally, k-fold cross-validation is utilized to train the models on benchmark datasets, and the performance of the models is compared on independent datasets. The study’s limitation arises from the exclusive use of the CNN model as the sole deep learning approach, without conducting a performance comparison with other deep learning models, which constrains the evaluation of the proposed model’s effectiveness.

In Feng et al. (2022), a novel and highly efficient method, called ME-ACP, is introduced, employing multi-view neural networks combined with an ensemble model for the identification of anticancer peptides. Initially, residue-level and peptide-level features are incorporated using ensemble models based on lightGBMs to yield preliminary results. Subsequently, the outputs of these lightGBM classifiers are fed into a hybrid deep neural network (HDNN) to accurately identify ACPs. The limitation of this study stems from the fact that while the performance of various deep learning and machine learning models has been comprehensively compared, the effectiveness of the proposed model, named ME-ACP, has not been evaluated in terms of model complexity and computational performance, which restricts the assessment of its overall effectiveness.

In Park et al. (2022), the construction of expansive non-repetitive training and independent datasets for the study of anticancer peptides is undertaken. Through the utilization of the training dataset, an extensive exploration of diverse feature encodings is conducted, leading to the development of corresponding models employing seven distinct conventional classifiers. Subsequently, a subset of encoding-based models is carefully chosen for each classifier based on their performance metrics. The predicted scores obtained from these selected models are concatenated and further trained using a CNN. The resulting predictor, known as MLACP 2.0, is thus established. Although this study comprehensively compares the performance of various machine learning models, it presents a limitation in terms of the evaluation of the model’s effectiveness because only the CNN model is considered as the deep learning model, and it is applied to a single dataset.

In Liu, Li & Chen (2022), a cutting-edge deep learning model named AntiMF is introduced, which incorporates a multi-view mechanism based on diverse feature extraction models. Comparative analysis reveals that the proposed model outperforms existing methods in predicting anticancer peptides. By employing an ensemble learning framework to extract representations, AntiMF effectively captures multidimensional information, thereby enhancing the comprehensiveness of model representation. In this study, despite conducting comprehensive experiments with different models and datasets, the high accuracy rates achieved have not been substantiated with metrics such as precision and recall. Therefore, in the presence of class imbalance in the dataset, a consistent interpretation of the model’s performance cannot be made. Additionally, the superior results obtained with the deep learning models used are not supported by evidence indicating whether the models are exposed to the over-fitting problem, thus posing a significant constraint on the assessment of the model’s effectiveness.

Ghulam et al. (2022) introduces a novel method named ACP-2DCNN, which utilizes deep learning techniques to improve the prediction accuracy of anticancer peptides. In both the training and prediction stages, crucial features are extracted using dipeptide deviation from expected mean (DDE), while a two-dimensional convolutional neural network (2D CNN) is employed. In this study, despite the use of two different datasets, the focus has been on a single feature selection technique and a single deep learning model. The absence of a comparison of the performance of the proposed methods with other models creates a limitation in assessing the model’s performance. Furthermore, when examining the training and test loss curves of the proposed model, it is observed that the model is faced to over-fitting problem, which raises concerns about the accuracy of the model’s performance.

In Alsanea et al. (2022), a robust framework is developed for the accurate identification of ACPs. The approach incorporates four distinct hypothetical feature encoding mechanisms, namely amino acid, dipeptide, tripeptide, and an enhanced version of pseudo amino acid composition, effectively capturing the motif characteristics of the target class. Additionally, principal component analysis (PCA) is employed for feature selection, focusing on identifying optimal, deep, and highly variable features. Given the diverse nature of the learning process, a range of algorithms is employed to determine the optimal operational method. Empirical investigations reveal that the support vector machine with hybrid feature space demonstrates superior performance. The proposed framework achieves an accuracy of 97.09% and 98.25% on the benchmark and independent datasets, respectively. In this study, although different feature extraction methods are applied on two different datasets, the models used in the learning phase are limited to traditional machine learning methods. Class imbalance issues in the datasets are not addressed through any preprocessing methods, and the results obtained are not supported by precision and recall metrics, which would reveal the impact of this imbalance. Therefore, the high results obtained may tend to be derived from examples where the class label is dense, indicating a potential problem of over-fitting.

In Ahmed et al. (2021), researchers propose a novel multi-headed deep convolutional neural network model, named ACP-MHCNN, designed to extract and integrate discriminative features from various information sources interactively. The model proficiently captures sequence, physicochemical, and evolutionary-based features for identifying ACPs using diverse numerical representations of peptides while effectively managing parameter overhead. Through rigorous experiments involving cross-validation and an independent dataset, the study demonstrates that ACP-MHCNN significantly outperforms other models in terms of ACP identification on the employed benchmarks. ACP-MHCNN achieves a remarkable improvement over the state-of-the-art model, surpassing it by 6.3% in accuracy, 8.6% in sensitivity, 3.7% in specificity, 4.0% in precision, and 0.20 in Matthews correlation coefficient (MCC) for predicting anticancer peptides. In this study, multiple feature extraction methods and combinations thereof have been applied on three different datasets. However, the issue of class imbalance has not been addressed in a similar manner, and the model training has been confined to a single model. While class imbalance can potentially lead to over-fitting in the model, no evidence of whether the model is overfitted the data has been found.

In Wu et al. (2019), a computational model for predicting therapeutic peptides (PTPD) is introduced, leveraging deep learning and Word2Vec techniques. The model obtains representation vectors for all k-mers through Word2Vec, considering the co-existence information among k-mers. Subsequently, the original peptide sequences are partitioned into k-mers using the windowing method. The embedding vector obtained from Word2Vec is used to map the peptide sequences to the input layer. Feature maps are constructed by applying three types of filters in the convolutional layers, accompanied by dropout and max-pooling operations. These feature maps are then concatenated into a fully connected dense layer, incorporating rectified linear units (ReLU) and dropout operations to prevent overfitting of PTPD. Classification probabilities are generated using a sigmoid function. The performance of PTPD is evaluated on two distinct datasets: an independent anticancer peptide dataset and a virulent protein dataset, achieving accuracies of 96% and 94%, respectively. In this study, the issue of class imbalance has not been addressed in a similar manner, and the model training has been limited to a single model and a single feature extraction technique. Additionally, it is not clear whether the proposed model is overfitted the data. Furthermore, despite the claim of the model’s effectiveness, no runtime analysis has been conducted.

In Yu et al. (2020), a thorough analysis is conducted on three fundamental deep learning architectures: convolutional, recurrent, and convolutional-recurrent networks, for distinguishing between ACPs and non-ACPs. The recurrent neural network featuring bidirectional long short-term memory cells is observed to outperform other architectural designs. Using the proposed model, a sequence-based deep learning tool named DeepACP is developed to effectively assess the probability of a peptide exhibiting anticancer activity. In this study, although different deep learning architectures are used, the research has been limited to a single dataset. Additionally, the models are fed with raw data without any feature extraction step.

In Sun et al. (2022), ACPNet is introduced as a novel deep learning-based model specifically crafted for discriminating between anticancer peptides and non-anticancer peptides (non-ACPs). ACPNet incorporates three distinct sources of peptide sequence information, including peptide physicochemical properties and auto-encoding features, integrated into the model’s training process. ACPNet adopts a hybrid architecture that combines fully connected networks with recurrent neural networks, leveraging the unique strengths of each approach. Evaluation of ACPNet on the ACPs82 dataset reveals noteworthy improvements, such as a 1.2% increase in accuracy, a 2.0% enhancement in F1-score, a 7.2% boost in recall, and well-balanced results in terms of the Matthews correlation coefficient. In this study, different feature extraction techniques are applied to various datasets. The feature space is expanded in this way with distinct techniques, and it is then fed into the proposed deep learning model and traditional machine learning algorithms. Additionally, it is evaluated in terms of precision and recall values, but the limited number of instances in the datasets do not allow the feature space to be adequately developed, resulting in limited success.

In Akbar et al. (2022), a FastText-based word embedding approach is employed to represent each peptide sample using a skip-gram model. The descriptors of peptide embeddings are extracted, and a deep neural network (DNN) model is applied to effectively discriminate antimicrobial cyclic peptides. Optimizing the DNN model’s parameters results in impressive accuracies of 96.94%, 93.41%, and 94.02% when utilizing training, alternate, and independent samples, respectively. The proposed cACP-DeepGram model demonstrates superior performance, exhibiting approximately 10% higher prediction accuracy compared to existing predictors. This suggests that the cACP-DeepGram model holds great promise as a reliable tool for scientists and can significantly contribute to academic research and drug discovery endeavors. In this study, although two different datasets are used, a single feature selection technique and classification model are employed. While it is proven that the model does not overfit the dataset it is trained on, its performance is limited because it is evaluated using only one learning model. Additionally, it is unclear whether the obtained success is due to the model used or the feature extraction technique.

## Models

Before diving into specific methodologies, it is essential to understand the foundations of word embedding models and their deep learning counterparts. Thus, this section offers an extensive exposition of word embedding models and deep learning methodologies that have been proposed and advanced specifically for the task of ACP classification.

### Word2Vec word embedding model

Word2Vec (Mikolov et al., 2013) is a word embedding model widely employed in natural language processing. It operates by generating dense vector representations for words in a continuous vector space. The model processes input text (typically a vast corpus) to understand and represent words as vectors based on the context in which they appear. Word2Vec comprises multiple layers, including an input layer, hidden layer(s), and an output layer. The input layer accepts word sequences from the corpus, while the hidden layer(s) learn to capture the contextual relationships between words. The output layer then predicts which words are most likely to appear near a given target word. The model incorporates various parameters that can be adjusted, such as the dimensionality of the word vectors, the window size (*i.e.,* the number of adjacent words considered for context), and the number of negative samples employed during training.

**Figure 1:**
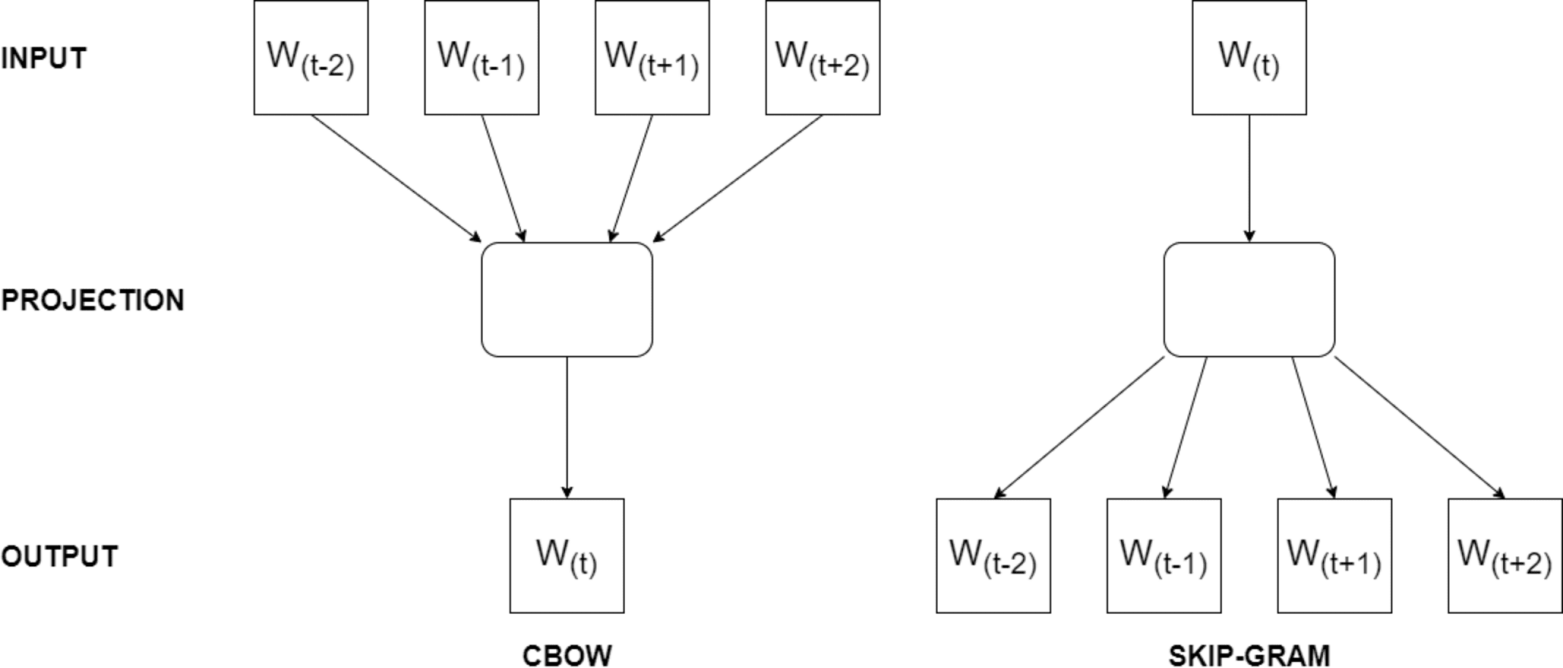
The architecture of Word2Vec models.

Word2Vec’s working logic revolves around optimizing the word vectors by iteratively predicting neighboring words given a target word using techniques like skip-gram or continuous bag-of-words (CBOW). The model updates the word vectors using stochastic gradient descent optimization during training. For instance, when the skip-gram model is trained on the sentence ‘I enjoy programming in Python,’ it learns to predict neighboring words for a target word. If we choose ‘programming’ as the target, it predicts words like ‘I,’ ‘enjoy,’ ‘in,’ and ‘Python’ as context words because they frequently appear within a short distance of ‘programming’ in the training data. On the other hand, CBOW model, when trained on the same sentence, it learns to predict the target word ‘programming’ based on the context words within a two-word window, such as ‘I,’ ‘enjoy,’ ‘in,’ and ‘Python,’ as they frequently co-occur in the training data. In Fig. 1, the architecture of Word2Vec training models are presented.

### Global vectors for word representation

Global Vectors for Word Representation (GloVe) (Pennington, Socher & Manning, 2014) is an unsupervised learning algorithm for obtaining word representations. It works by capturing the semantic and syntactic relationships between words in large corpora of text. The core idea behind GloVe is to use word co-occurrence statistics to learn the word vectors. Essentially, it tries to find vectors for words that encode meaningful information about their relationship with other words. This is done through the analysis of the frequency of word co-occurrences in a given text corpus. GloVe’s architecture is based on a matrix factorization approach. It starts by constructing a word co-occurrence matrix, which records how often words appear together in the same context within the corpus. The algorithm then factorizes this matrix to generate word vectors. The resulting vectors aim to represent words in a continuous vector space, where the distance between vectors encodes information about the similarity and relationships between words.

**Figure 2:**
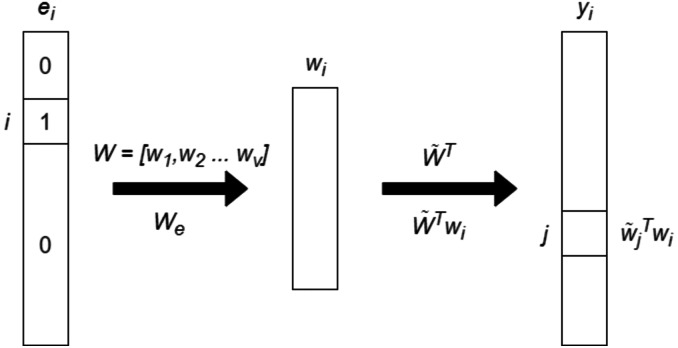
The basic architecture of GloVe model.

GloVe and Word2Vec are both word embedding models but differ in their approach to learning word representations. Word2Vec uses neural networks to predict the context of a word (Continuous Bag of Words or Skip-gram models), while GloVe focuses on leveraging global word co-occurrence statistics. In details, Word2Vec can capture complex word relationships, including analogies and syntactic structures while GloVe tends to emphasize global word relationships more effectively. In addition, Word2Vec may perform better on rare words, whereas GloVe often excels at representing common words. In Fig. 2, the basic architecture of GloVe model is presented.

### FastText word embedding model

FastText (Bojanowski et al., 2017) is a word embedding model designed for natural language processing tasks. It represents words as continuous-valued vectors, capturing both semantic and syntactic information. FastText processes input text data in an unsupervised manner, employing a shallow neural network architecture with multiple layers to efficiently learn word embeddings. FastText differs from Word2Vec by its unique capability to generate word vectors for out-of-vocabulary words using subword information, breaking words into smaller units like character n-grams. This means FastText can handle a more extensive vocabulary, particularly in languages with rich morphological features, making it suitable for tasks involving a wide range of words and languages, while Word2Vec struggles with out-of-vocabulary words due to its word-level approach.

The model comprises an input layer, a hidden layer, and an output layer. In the input layer, text data undergoes transformation into n-gram character sequences, capturing subword information. These subword sequences then pass through the hidden layer, applying a non-linear transformation to generate feature representations. Finally, the output layer predicts the word’s context based on the learned representations. By incorporating subword information, FastText adeptly captures morphological variations and handles out-of-vocabulary words more effectively than traditional word embeddings.

**Figure 3:**
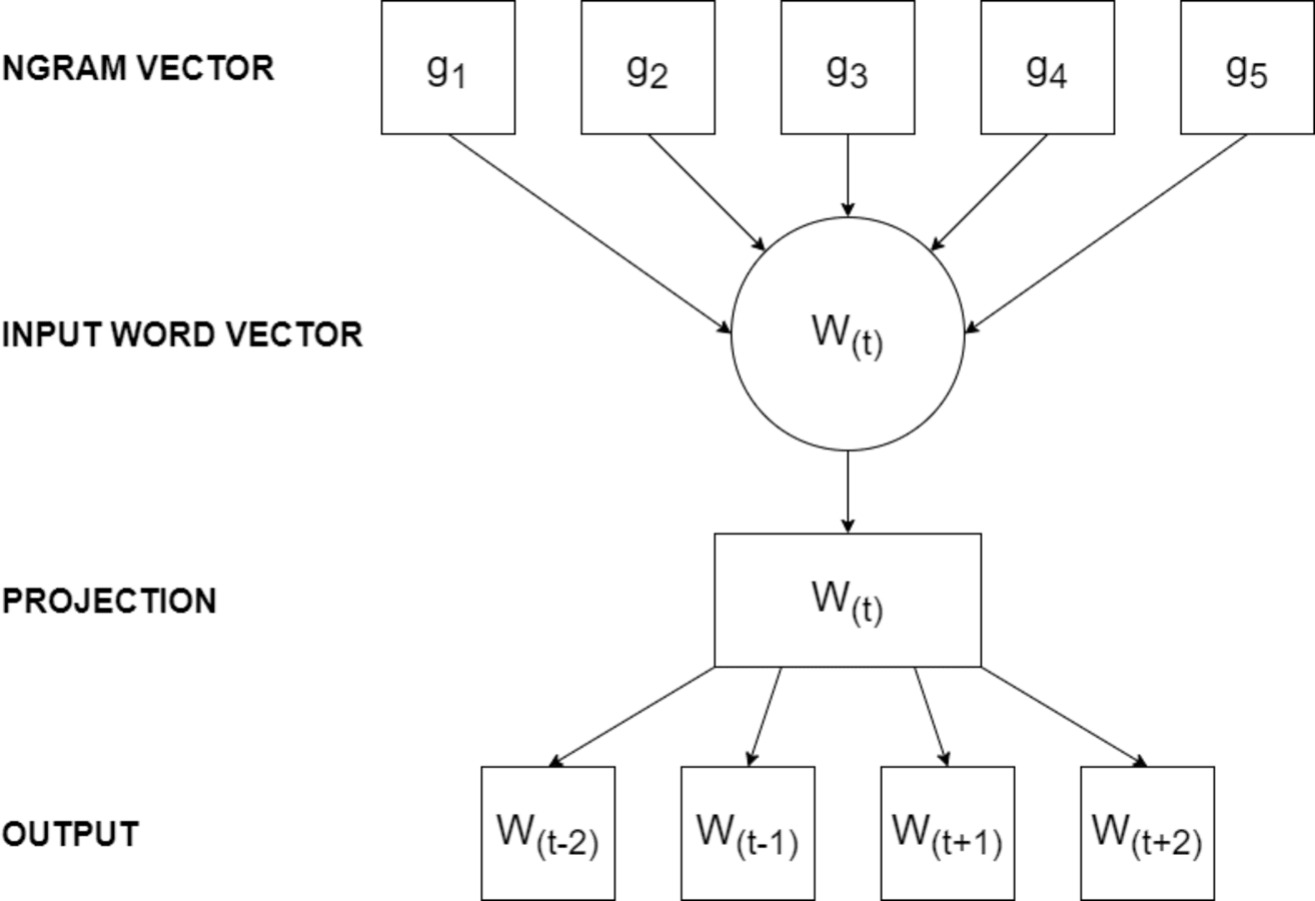
The architecture of FastText model.

The FastText model has various parameters influencing its performance. The embedding dimension, determining the size of word vectors, stands out as a critical parameter. Additionally, the model allows adjustment of the learning rate, the number of training epochs, and the size of the context window. Figure 3 illustrates the architecture of the FastText training model.

### One-Hot-Encoding

One-Hot-Encoding (OHE) is a technique used to convert categorical data, such as words in a text corpus, into a numerical format suitable for machine learning models. One-Hot-Encoding is a simple and effective method for representing categorical data numerically. It works by creating a binary vector for each category (word in the case of natural language processing). Each vector has the same length as the total number of unique categories in the dataset. To encode a specific category, a 1 is placed in the position corresponding to that category, and 0s are placed in all other positions. These binary vectors are typically sparse, with mostly 0s and a single 1 at the position representing the specific word.

One-Hot-Encoding results in high-dimensional, sparse vectors, whereas Word2Vec, GloVe, and FastText generate dense, lower-dimensional word embeddings. In addition, One-Hot-Encoding does not capture semantic relationships between words, whereas the embedding models aim to represent words in a continuous vector space where distances encode semantic similarity. Word embedding models typically require less memory and storage compared to One-Hot-Encoding for large vocabularies. Furthermore, word embedding models generalize to unseen words by learning continuous representations, while One-Hot-Encoding treats each word as a unique and unrelated entity.

### Convolutional neural network

Convolutional neural network (CNN) is a deep learning model (LeCun et al., 1998) designed to extract features from input data, particularly in image and text analysis. It has demonstrated exceptional capabilities in various domains, including image classification, object detection, sentiment analysis, document classification, and text generation. CNN follows a hierarchical architecture with multiple layers, each performing specific operations to learn and recognize patterns.

In the case of text or image input, CNN processes the data through convolutional layers. These layers use convolution operations on localized regions of the input, referred to as receptive fields or filters, to extract relevant features. As the filters slide over the input, dot products are computed between their weights and input values, generating feature maps that capture different aspects of the data. CNN typically comprises an input layer, convolutional layers, pooling layers, fully connected layers, and an output layer. Convolutional layers perform feature extraction using filters with shared weights, allowing the network to learn spatial hierarchies of features. Pooling layers reduce the spatial dimensions of the feature maps while retaining salient information. Fully connected layers integrate the extracted features for predictions, and the output layer provides the final classification or regression output.

**Figure 4:**
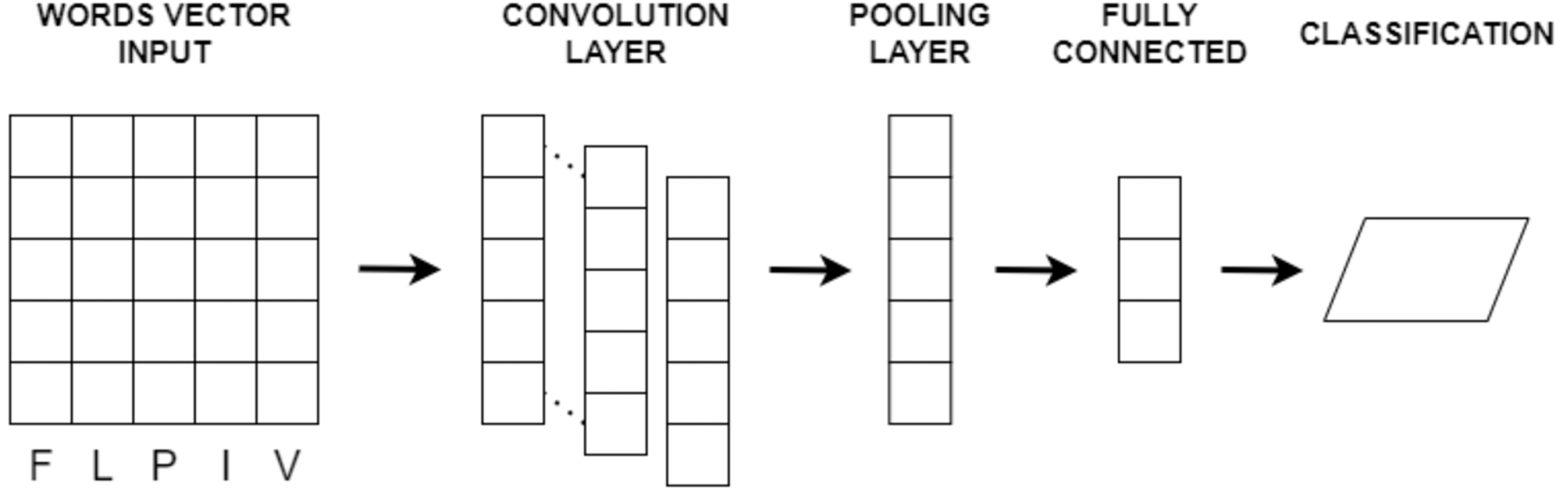
The basic CNN architecture.

Various parameters impact CNN performance, including the number and size of filters, convolutional operation stride, pooling region size, and activation functions. Hyperparameters like learning rate, regularization techniques, and optimization algorithms are crucial for effective CNN training. CNNs operate on the principles of local receptive fields and shared weights, automatically learning relevant features by applying filters across input data. Pooling layers subsequently reduce feature dimensionality while preserving discriminative information. The basic CNN architecture is depicted in Fig. 4.

### Long short term memory network

The long short term memory (LSTM) deep learning model (Hochreiter & Schmidhuber, 1997) is a type of recurrent neural network (RNN) designed to address the limitations of traditional RNNs in capturing and retaining long-term dependencies in sequential data.

LSTMs operate by incorporating a memory cell that selectively retains or forgets information over extended sequences. This memory cell stores and propagates information across time steps, enabling the network to capture long-term dependencies. The key innovation of LSTMs lies in the use of gating mechanisms, including input, forget, and output gates, which regulate the flow of information within the network. LSTMs take sequential data as input, such as text, time series, or other sequential data, with each element of the sequence represented as a vector. The LSTM processes the sequence one element at a time.

Comprising multiple layers, each with multiple LSTM units, LSTMs feature three main components within each unit: the input gate, the forget gate, and the output gate. These gates control information flow and memory cell updates. LSTM parameters include weights and biases associated with each gate and the memory cell, learned during training using backpropagation through time (BPTT) or other optimization algorithms, with the objective of minimizing a defined loss function.

**Figure 5:**
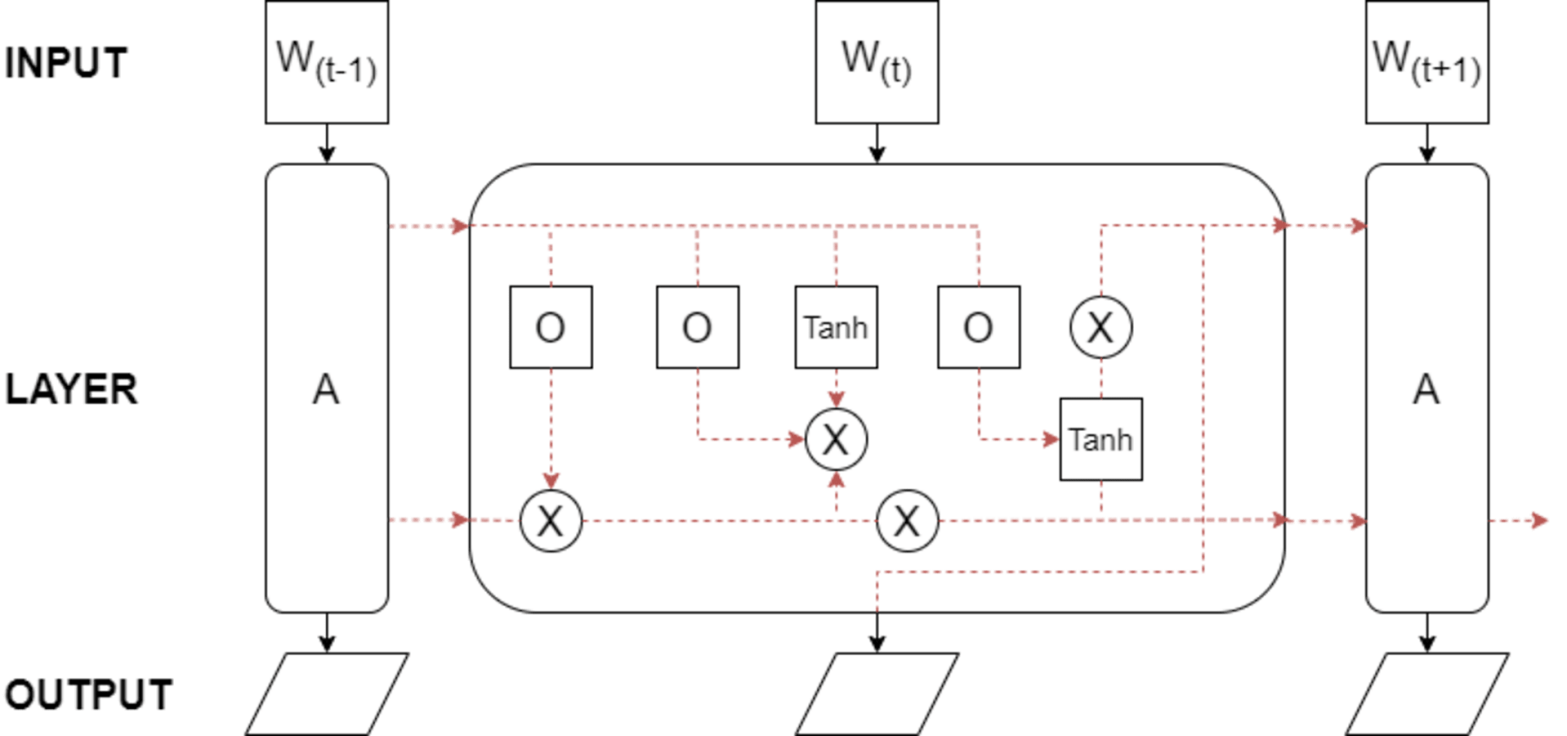
The basic LSTM architecture.

The performance of LSTM models depends on factors like network size and architecture, training data quality and size, choice of optimization algorithm, and model hyperparameters. LSTMs have found success in diverse domains, including natural language processing, speech recognition, machine translation, and time series analysis. The foundational architecture of LSTM is depicted in Fig. 5.

### Bidirectional long short term memory networks

The bidirectional long short term memory (BiLSTM) is a deep learning model designed for sequential data, capturing information from both past and future contexts (Graves, Jaitly & Mohamed, 2013). An extension of the traditional LSTM, the BiLSTM has gained popularity for its capability to consider context bidirectionally. It operates on input text formatted as sequential data, such as sentences or time series, and is commonly applied in tasks like sequence labeling, sentiment analysis, machine translation, and speech recognition. Enhancements to the BiLSTM’s performance can be achieved by incorporating techniques like attention mechanisms or additional layers. The input text is typically tokenized into individual units, such as words or characters, and then encoded as numerical vectors using methods like one-hot encoding or word embeddings.

**Figure 6:**
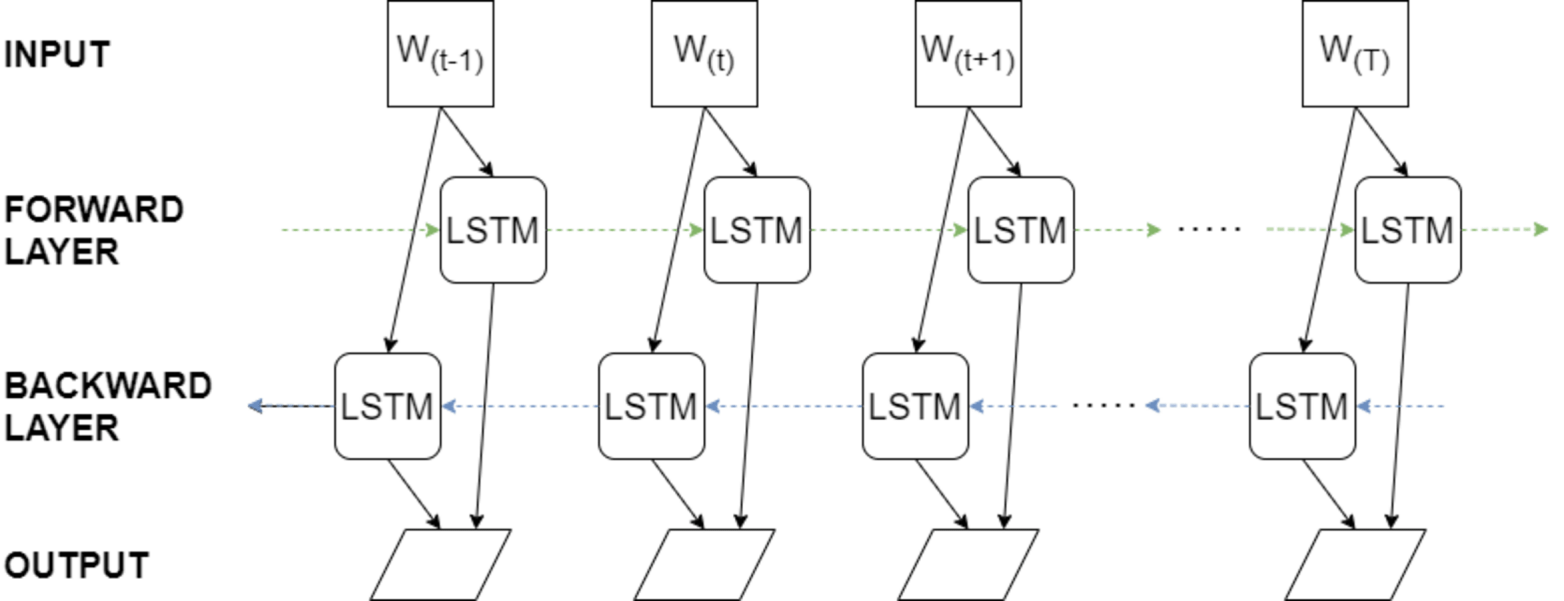
The basic BiLSTM architecture.

The BiLSTM model comprises multiple layers of LSTM units, each equipped with a memory cell and three primary gates: the input gate, the forget gate, and the output gate. These gates manage information flow and oversee the state of the memory cell. By integrating both forward and backward LSTM layers, the BiLSTM captures dependencies in the input sequence from both directions, enabling it to effectively grasp long-term dependencies and context. The model’s parameters include the number of LSTM units, the number of layers, the input and output dimensions, and the activation functions. Training the model involves backpropagation through time, where gradients are computed and used to update the weights of the LSTM units. The foundational architecture of BiLSTM is depicted in Fig. 6.

## Proposed Framework

In this section, datasets employed in this study and proposed methodology are introduced.

### Dataset

The ACPs250 dataset (Yu et al., 2020), a primary data source in this study, comprises a total of 500 peptide instances. Specifically, it includes 250 peptides labeled as “anticancer” and an equal number labeled as “non-anticancer.” This balanced dataset plays a crucial role in training and evaluating classification models to distinguish between these two distinct classes. The inclusion of 250 anticancer-labeled peptides and 250 non-anticancer-labeled peptides allows for a comprehensive analysis of their characteristic features, facilitating the development of effective predictive models. Leveraging the ACPs250 dataset, this study aims to contribute to anticancer peptide research by exploring discriminative patterns and underlying properties distinguishing anticancer peptides from their non-anticancer counterparts. For detailed statistics and dataset content, refer to Tables 1, 2, and 3.

**Table 1:** Statistics of the datasets.

**Dataset**	**Positive**	**Negative**	**Total**
ACPs250	250	250	500
Independent	150	150	300

**Table 2:** Content of the ACPs250 dataset.

**Sample**	**Content**	**Label**
1	KWKLFKKIEKVGQNIRDGIIKAGPAVA	0
2	FLPAIVGAAAKFLPKIFCAISKKC	0
...	...	...
499	FLPIVTNLLSGLL	1
500	GALRGCWTKSYPPKPCK	1

**Table 3:** Content of the independent dataset.

**Sample**	**Content**	**Label**
1	AAKKWAKAKWAKAKKWAKAA	0
2	AAVPIVNLKDELLFPSWEALFSGSE	0
...	...	...
299	VTSWSLCTPGCTSPGGGSNCSFCC	1
300	YVPLPNVPQPGRRPFPTFPGQG	1

The Independent dataset (Chen et al., 2016), consisting of 300 peptide sequences, serves as another critical data source in this research endeavor. It encompasses 150 peptides labeled as “anticancer” and an equal number of peptides labeled as “non-anticancer”. This dataset offers a unique and separate collection of peptides, distinct from the ACPs250 dataset, to validate and evaluate the generalization capability of the developed models. The inclusion of 150 anticancer-labeled peptides and 150 non-anticancer-labeled peptides in the Independent dataset enables a comprehensive assessment of the model’s performance on unseen data, thereby providing insights into its robustness and reliability. By incorporating the Independent dataset, this study aims to strengthen the credibility and applicability of the proposed models in the realm of anticancer peptide prediction and classification. In Tables 1 and 3, the details of Independent dataset are presented.

### Proposed methodology

**Figure 7:**
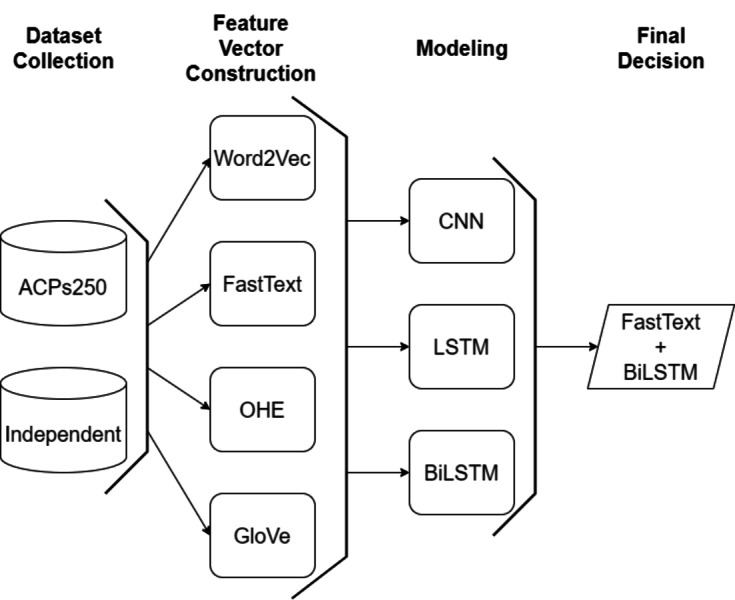
A general flow chart of the proposed model.

**Figure 8:**
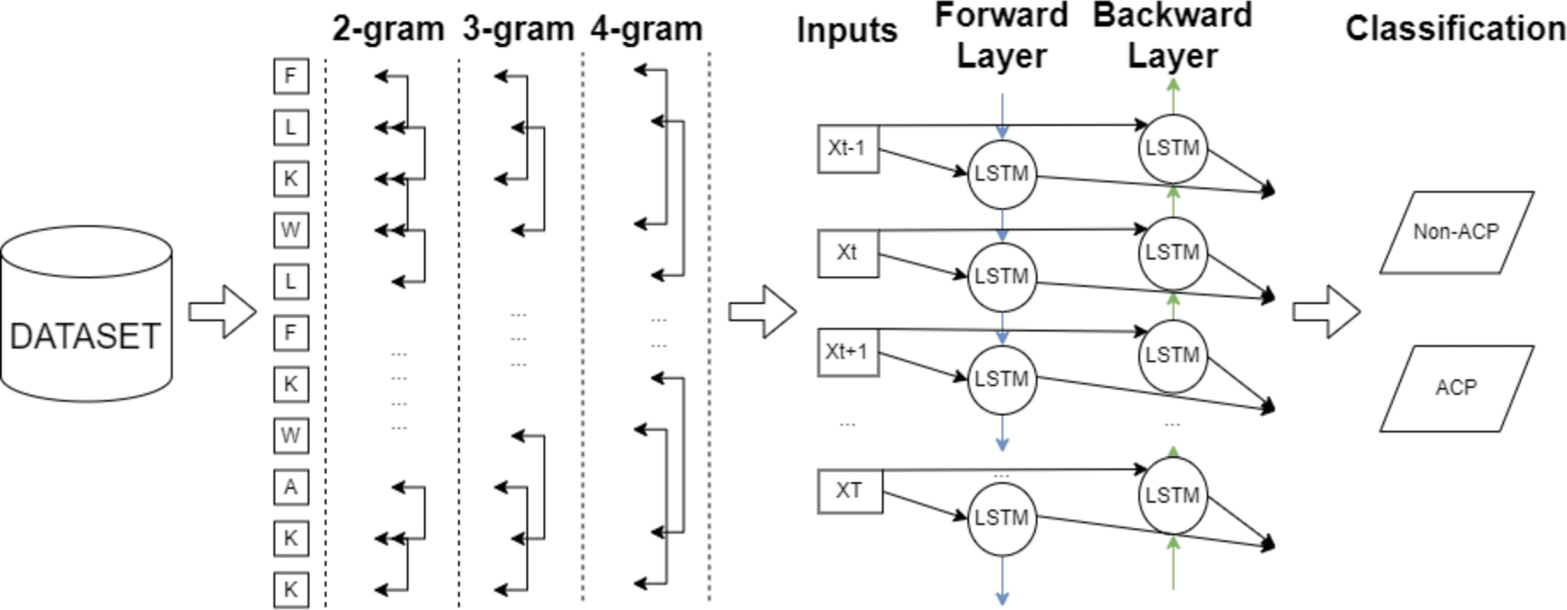
The architecture of consolidated FastText+BiLSTM framework.

In this study, an efficient classification framework is proposed for the purpose of classifying anticancer peptides with the aid of word embedding models and deep learning methodologies. After gathering widely applied datasets, character-based feature vectors are extracted from peptide sequences on ACPs250, and Independent datasets employing two versions of Word2Vec, GloVe, FastText, and One-Hot-Encoding models. The Word2Vec model encompasses both skip-gram and CBOW techniques to perform the word embedding process. In the FastText model, various n-gram units ranging from 2-gram to 4-gram are considered during the embedding process. After constructing the feature vector for each sample on both datasets, the processed datasets are inputted into CNN, LSTM, and BiLSTM deep learning algorithms. The general flowchart of the proposed framework is depicted in Fig. 7. As shown in Fig. 7, the final decision is ensured by the combination of the FastText and BiLSTM models as a result of extensive experiments. The architecture of the consolidated FastText+BiLSTM framework is presented in Fig. 8.

During the model construction phase, various parameters are employed. The number of layers is varied between 1 and 5, enabling the exploration of models with different layer configurations. Dropout regularization is applied at rates of 0.1, 0.2, 0.3, 0.4, and 0.5 to mitigate overfitting problems. Batch sizes of 16, 32, 64, 96, 128, and 192 are also tested. Some models include max pooling and batch normalization to enhance classification success. Various activation functions, including sigmoid, softmax, and relu, are examined individually or in combination. Different learning rates of 0.1, 0.01, 0.001, and 0.0001 are employed to address the issue of overfitting. Loss types, namely binary crossentropy and categorical crossentropy, are employed to achieve optimal results in the classification task. Through the incorporation of these diverse parameters, the performance of the models is thoroughly analyzed, leading to the identification of the most successful models for each dataset.

In details, the CNN model consists of several layers and parameters designed to effectively capture and classify the peptide sequences. The CNN architecture begins with an embedding layer that employs Word2Vec, GloVe, FastText, and One-Hot-Encoding methods, separately in order to convert peptide sequences into a vector space. This layer maps peptide sequences to a continuous vector space, enabling the capture of semantic meaning and relationships. Following the embedding layer, the model utilizes a 64-unit CNN layer with a rectified linear unit (ReLU) activation function. This layer performs convolutions on the embedded peptide vectors, extracting relevant features and patterns. Subsequently, dropout regularization with a rate of 0.3 is applied twice to mitigate the overfitting issue. Dropout randomly deactivates a portion of the neurons during training, promoting model generalization. Finally, a softmax activation function is employed in the dense layer to produce probabilistic predictions. This layer computes the probability distribution over the two classes (“anticancer” and “non-anticancer”) based on the learned features. The softmax activation ensures that the predicted probabilities sum up to 1, enabling the interpretation of class probabilities. The model is compiled with the binary crossentropy loss function, suitable for binary classification tasks, and the Adam optimizer with a learning rate of 0.01. The Adam optimizer adapts the learning rate dynamically based on the estimated gradients, facilitating efficient convergence and optimization.

The LSTM model starts with an embedding layer that utilizes Word2Vec, GloVe, FastText, and One-Hot-Encoding methods, separately for the purpose of transforming the peptide sequences into continuous vector representations, thence, capturing their semantic meaning and contextual information. After the embedding layer, the model employs an LSTM layer featuring 32 units and a rectified linear unit (ReLU) activation function. LSTM, a variant of recurrent neural networks (RNNs), proves effective in capturing prolonged dependencies within sequential data. This layer processes the embedded peptide vectors, preserving crucial temporal information. To mitigate overfitting and bolster generalization, two dropout layers, each with a dropout rate of 0.3, are integrated into the model. Dropout selectively deactivates a portion of neurons during training, encouraging the model to depend on a more diverse and robust set of features.

Finally, a softmax activation function is applied in the dense layer to compute class probabilities for the classification task. The model is compiled using the binary-crossentropy loss function, well-suited for binary classification problems, and the Adam optimizer with a learning rate of 0.01. This configuration ensures efficient training and convergence of the model.

Subsequent to the preceding layers, a BiLSTM layer with 64 units is engaged to capture sequential dependencies within the text data by processing input in both forward and backward directions. The bidirectional property of the BiLSTM enables it to comprehend context from both preceding and subsequent tokens, thereby adeptly capturing extensive temporal dependencies. Following this BiLSTM layer, a dropout regularization technique is implemented with a rate of 0.2. Following this, a BiLSTM layer with 32 units is introduced, succeeded by an additional dropout layer with a rate of 0.2. This architectural configuration facilitates the model in discerning more intricate relationships and capturing finer nuances within the text data.

Subsequently, a BiLSTM layer with 16 units and an accompanying dropout layer with a rate of 0.2 are incorporated. These layers further enhance the model’s capacity to capture intricate patterns and subtle details inherent in the text data. The model culminates in a dense layer employing the sigmoid activation function, serving as the final output and predicting class probabilities (*e.g.*, “anticancer” or “non-anticancer”). Training of the model involves utilizing the binary-crossentropy loss function to minimize the disparity between predicted class probabilities and true class labels. The Adam optimizer is employed for weight updates during training, adapting the learning rate (set to 0.01) based on gradient information, facilitating efficient model convergence.

In summary, this model leverages a series of BiLSTM layers, dropout regularization, and embedding layers to adeptly capture complex relationships and temporal dependencies inherent in text data.

## Experiment Results

In this section, the consolidation of word embedding models and deep learning techniques are evaluated in terms of different evaluation and performance metrics for the purpose of anticancer peptides classification task. ACC for accuracy, SEN for sensitivity, SPE for specificity, MCC for Matthew correlation coefficient, and AUC for area under the curve are abbreviated as evaluation metrics in the tables to demonstrate the classification success of each model and the contribution of our study. These values are given in the table as % ratio. All models are run on Google Colab environment provided free GPU usage by Google. Experiments are accomplished using 80% training and 20% test of data with repeated holdout method. It is applied 10 times on each data set. The following abbreviations are employed for the combination of word embedding techniques and deep learning methods: GL+CNN: consolidation of GloVe and CNN, GL+LSTM: combination of GloVe and LSTM, GL+BiLSTM: combination of GloVe and BiLSTM, OHE+CNN: combination of One-Hot-Encoding embedding and CNN, OHE+LSTM: combination of One-Hot-Encoding embedding and LSTM, OHE+BiLSTM: combination of One-Hot-Encoding embedding and BiLSTM, WS+CNN: combination of Skip-gram version of Word2Vec and CNN, WC+CNN: combination of CBOW version of Word2Vec and CNN, WS+LSTM: combination of Skip-gram version of Word2Vec and LSTM, WC+LSTM: combination of CBOW version of Word2Vec and LSTM, WS+BiLSTM: combination of Skip-gram version of Word2Vec and BiLSTM, WC+BiLSTM: combination of CBOW version of Word2Vec and BiLSTM, FT+CNN: combination of FastText and CNN, FT+LSTM: combination of FastText and LSTM, FT+BiLSTM: combination of FastText and BiLSTM. FastText word embbedding model employed in the experiments are asssesed by varying number of n-gram in the range of 2–4. The best accuracy percentages are represented in bold letters.

As a first attempt, we analyse the effect of all aforementioned embedding models and deep learning techniques on two different datasets in terms of various evaluation metrics. In Table 4, the experiment results of the combination of embedding techniques namely, GloVe, OHE, and deep learning models are presented in ACP250 dataset. It is observed that GloVe and OHE as embedding models yield the best performance when fed into deep learning models, with BiLSTM model achieving accuracy rates of approximately 81.99% and 87.99%, respectively. On the other hand, when GloVe is fed into a CNN model, it achieves approximately 7% better performance compared to the LSTM model. Conversely, OHE inputted into the LSTM model results in approximately an 8% increase in classification performance compared to the CNN model. Moreover, the GL+BiLSTM model demonstrates the ability to detect positive classes at 81.48% and negative classes at 82.60%, respectively when examining the metrics of sensitivity and specificity. The OHE+BiLSTM model, on the other hand, has been able to detect positive classes with a sensitivity value of 88.88% and negative classes with a specificity score of 87.95%, respectively. The results presented in Table 4 demonstrate the most successful outcomes obtained after hyper-parameter tuning that is represented for ACP250 dataset as Tables S1 and S2 in supplemental material. In the tables providing information about all hyperparameter settings, six results with the accuracy values out of 100 trials are listed. The parameter details listed in the tables include epoch size, dropout rate, batch size, learning rate, and activation function.

**Table 4:** Experiment results of GloVe and OHE embedding models with deep learning algorithms for ACP250 dataset. The best accuracy percentages are represented in bold letters.

**MODELS**	**ACC**	**SEN**	**SPE**	**MCC**	**AUC**
GL+CNN	81.00	89.00	81.73	71.95	80.31
GL+LSTM	74.00	76.29	74.82	71.48	73.06
GL+BiLSTM	**81.99**	81.48	82.60	82.93	82.04
OHE+CNN	75.00	76.29	74.67	72.28	73.14
OHE+LSTM	83.99	85.62	88.13	81.59	84.38
OHE+BiLSTM	**87.99**	88.88	87.95	85.84	87.92

In Table 5, the experimental outcomes of the combination of embedding techniques namely, GloVe, OHE, and deep learning models are presented in Independent dataset. It is observed that GloVe and OHE as embedding models perform the best classification success when fed into deep learning models, with BiLSTM model achieving accuracy rates of approximately 85.93% and 82.81%, respectively. On the other hand, when GloVe is fed into a LSTM model, it achieves nearly 2% better performance compared to the CNN model. Conversely, OHE inputted into the CNN model results in approximately an 18% increase in classification performance compared to the LSTM model. Furthermore, the GL+BiLSTM model demonstrates the ability to detect positive classes at 88.92% and negative classes at 93.54%, respectively when examining the metrics of sensitivity and specificity. The OHE+BiLSTM model, on the other hand, has been able to detect positive classes with a sensitivity value of 86.13% and negative classes with a specificity score of 84.74%, respectively. The results presented in Table 5 demonstrate the most successful outcomes obtained after hyper-parameter tuning that is represented for Independent datasetas Tables S3 and S4.

**Table 5:** Experiment results of GloVe and OHE embedding models with deep learning algorithms for independent dataset. The best accuracy percentages are represented in bold letters.

**MODELS**	**ACC**	**SEN**	**SPE**	**MCC**	**AUC**
GL+CNN	82.81	90.90	74.19	66.24	82.55
GL+LSTM	84.37	79.69	88.57	72.59	84.87
GL+BiLSTM	**85.93**	88.92	93.54	72.87	86.16
OHE+CNN	75.16	85.23	82.54	77.08	84.19
OHE+LSTM	57.81	58.36	67.46	51.17	60.09
OHE+BiLSTM	**82.81**	86.13	84.74	78.09	82.35

In Table 6, it is observed that the model employing the WS embedding algorithm, in conjunction with the CNN deep learning architecture, manifests the lowest performance with an accuracy of 57.99%. On the other hand, the model utilizing the WC embedding algorithm and CNN architecture demonstrates a relatively higher accuracy value of 74.00%. Conversely, the integration of the WS embedding algorithm with the LSTM architecture yields a superior accuracy value of 75.99%, while the utilization of the WC with the LSTM architecture results in a slightly lower accuracy value of 73.00%. Notably, the consolidation of WS embedding algorithm and BiLSTM exhibits a superior performance with accuracy of 87.50%. Furthermore, the model utilizing the WC with the BiLSTM architecture showcases the best classification success exhibiting 90.00% of accuracy. Furthermore, the WC+BiLSTM model demonstrates the ability to detect positive classes at 92.50% and negative classes at 87.50%, respectively when examining the metrics of sensitivity and specificity. The results presented in Table 6 demonstrate the most successful outcomes obtained after hyper-parameter tuning that is represented for ACP250 datasetas Table S5.

**Table 6:** Experiment results of Word2Vec embedding model with deep learning algorithms for ACPs250 dataset. The best accuracy percentages are represented in bold letters.

**MODELS**	**ACC**	**SEN**	**SPE**	**MCC**	**AUC**
WS+CNN	57.99	59.95	57.04	16.03	62.37
WC+CNN	74.00	72.34	79.56	41.89	81.23
WS+LSTM	75.99	72.68	81.23	50.96	85.79
WC+LSTM	73.00	71.42	74.23	47.78	81.06
WS+BiLSTM	87.50	86.12	89.67	76.13	93.45
WC+BiLSTM	**90.00**	92.50	87.50	79.08	94.25

An evident observation emerges from our experimentation, indicating that the Word2Vec Skip-gram (WS) embedding algorithm demonstrates a notably reduced performance in CNN and BiLSTM models compared to the Word2Vec Continuous Bag of Words (WC) word embedding model. Notably, in the context of the LSTM model, the WS approach yields a superior accuracy value compared to the WC version of Word2Vec. Conversely, the Continuous Bag of Words (CBOW) method achieves a higher accuracy value in CNN and BiLSTM models while exhibiting relatively suboptimal performance in the LSTM framework. This empirical finding strongly suggests that the WC technique excels at capturing general word relationships. In conclusion, weighing the accuracy values and the specific model architectures employed, the integration of the WC embedding algorithm with the BiLSTM model emerges as the optimal choice, delivering the highest attainable accuracy result.

In Table 7, it is seen that the model combination of WS embedding algorithm with the CNN architecture perform the lowest accuracy score with 57.81%. In comparison, the model employing the WC embedding algorithm with the CNN architecture achieves a significantly higher accuracy value of 84.25%. This suggests that the WC method is more effective in capturing word relationships and performing well when combined with the CNN model. Moving on to the LSTM model, the combination of the WS embedding algorithm with the LSTM architecture yields an accuracy value of 88.75%, which is higher than the WS + CNN model. Similarly, the model utilizing the WC embedding algorithm with the LSTM architecture achieves an accuracy value of 86.51%. These results indicate that both version of Word2Vec embedding algorithms perform well in the LSTM models compared to the CNN method combinations. WS approach exhibiting slightly higher accuracy in comparison with WC model when consolidated with LSTM architecture. Considering the BiLSTM models, the model incorporating the WS embedding algorithm achieves an accuracy value of 89.06%, while the model utilizing the WC embedding algorithm achieves the highest accuracy value of 92.31%. These findings suggest that the combination of the WC embedding algorithm with the BiLSTM architecture yields the best performance among all the models examined. Moreover, the WC+BiLSTM model demonstrates the ability to detect positive classes at 93.79% and negative classes at 91.52%, respectively when examining the metrics of sensitivity and specificity. The results presented in Table 7 demonstrate the most successful outcomes obtained after hyper-parameter tuning that is represented for Independent dataset as Table S6

**Table 7:** Experiment results of Word2Vec embedding model with deep learning algorithms for independent dataset. The best accuracy percentages are represented in bold letters.

**MODELS**	**ACC**	**SEN**	**SPE**	**MCC**	**AUC**
WS+CNN	57.81	55.29	60.33	14.17	57.98
WC+CNN	84.25	80.10	68.36	52.47	83.05
WS+LSTM	88.75	89.25	88.31	77.48	93.65
WC+LSTM	86.51	82.62	90.74	72.83	91.25
WS+BiLSTM	89.06	88.88	89.37	77.65	94.05
WC+BiLSTM	**92.31**	93.79	91.52	84.62	96.55

In summary, a meticulous analysis of the provided table unveils that the choice of the Word2Vec embedding algorithm and the deep learning architectural framework exert a profound impact on the overall accuracy of the models under investigation. Notably, the Word2Vec Continuous Bag of Words (WC) embedding algorithm demonstrates a proclivity for superior performance within CNN models. Meanwhile, in the domain of LSTM models, both the Word2Vec Skip-gram (WS) and WC approaches manifest commendable proficiency. However, when applied to the BiLSTM models, the WC embedding algorithm emerges as the undisputed champion, exhibiting the highest attainable accuracy scores. This accomplishment underscores its exceptional aptitude for capturing intricate sequential data relationships. It is of paramount significance to underline that the juxtaposition of the results from Tables 6 and 7 illuminate a conspicuous trend that the adoption of the BiLSTM architectural framework synergizes remarkably well with the CBOW version of the Word2Vec model, resulting in a substantial elevation of the classification performance.

Upon examining the accuracy results of Table 8, it is observed that the model utilizing the FT(2) embedding algorithm with the CNN architecture achieves an accuracy of 88.99%. It is followed by the model incorporating the FT(3) embedding algorithm with the CNN architecture demonstrates a slightly lower accuracy value of 83.99%. The model employing the FT(4) embedding algorithm with the CNN architecture shows the lowest accuracy score with 57.99%. Moving on to the LSTM models, the combination of the FT(2) embedding algorithm with the LSTM architecture attains an accuracy of 72.00%. Additionally, the model utilizing the FT(3) embedding algorithm with the LSTM architecture achieves a higher accuracy value of 75.99%. However, the model incorporating the FT(4) embedding algorithm with the LSTM architecture shows the lowest accuracy value with 60.22%. Lastly, considering the BiLSTM models, the model utilizing the FT(2) embedding algorithm achieves an accuracy value of 82.99%. The combination of the FT(3) embedding algorithm with the BiLSTM architecture yields the highest accuracy with 92.50%. Conversely, the model incorporating the FT(4) embedding algorithm with the BiLSTM architecture demonstrates the lowest accuracy performance with 66.15% similar to the combination of FT(4) and other deep learning architectures. Moreover, the FT(3)+BiLSTM model indicates the ability to detect positive classes at 96.29% and negative classes at 82.60%, respectively when examining the metrics of sensitivity and specificity. The results presented in Table 8 demonstrate the most successful outcomes obtained after hyper-parameter tuning that is represented for ACP250 dataset as Table S7.

**Table 8:** Experiment results of FastText Embedding model including various N-grams with deep learning algorithms for ACPs250 dataset. The best accuracy percentages are represented in bold letters.

**MODELS**	**ACC**	**SEN**	**SPE**	**MCC**	**AUC**
FT(2)+CNN	88.99	86.27	89.75	77.63	93.51
FT(3)+CNN	83.99	80.58	87.42	67.29	90.20
FT(4)+CNN	57.99	55.79	59.83	15.74	60.29
FT(2)+LSTM	72.00	65.22	78.13	40.85	76.68
FT(3)+LSTM	75.99	70.34	82.49	49.32	82.17
FT(4)+LSTM	60.22	55.79	59.83	15.74	60.29
FT(2)+BiLSTM	82.99	79.68	86.34	70.01	90.01
FT(3)+BiLSTM	**92.50**	96.29	82.60	80.27	89.45
FT(4)+BiLSTM	66.15	55.79	59.83	15.74	60.29

Upon a meticulous examination of the outcomes, it is evident that the choice of FastText n-gram embedding algorithms and deep learning architectural designs exerts a significant influence on the overall model accuracy. The FT(2) embedding algorithm prominently demonstrates exceptional efficacy in the context of CNN models, consistently yielding elevated accuracy metrics across various n-gram iterations of the FastText model. Conversely, the FT(3) embedding algorithm consistently showcases superior performance, with the notable exception of the CNN architecture. Strikingly, the FT(4) embedding algorithm consistently registers the least favorable accuracy scores across all architectural configurations. In summation, the scrutiny of the presented table underscores the pivotal role played by the selection of the FastText n-gram embedding algorithm and the specific deep learning architectural framework in the attainment of precision in model outcomes. The FT(2) embedding algorithm emerges as a dependable choice tailored for the CNN model, while the FT(3) variant excels, especially in the BiLSTM model. Conversely, the FT(4) embedding algorithm, on average, exhibits relatively diminished effectiveness.

In Table 9, the accuracy results reveal important insights about the models that combine the FastText embedding algorithm with different deep learning architectures. The model utilizing the FT(2) embedding algorithm in conjunction with the CNN architecture demonstrates an accuracy value of 92.18%. The model incorporating the FT(3) embedding algorithm with the CNN architecture achieves a higher accuracy value of 93.75%. In contrast, the model employing the FT(4) embedding algorithm with the CNN architecture shows the lowest accuracy score of 57.81%. When considering the LSTM models, the combination of the FT(2) embedding algorithm with the LSTM architecture attains an accuracy value of 87.50%. Additionally, the model utilizing the FT(3) embedding algorithm with the LSTM architecture achieves a slightly higher accuracy value of 89.06%. However, the model incorporating the FT(4) embedding algorithm with the LSTM architecture demonstrates the lowest accuracy value of 62.33%. Turning attention to the BiLSTM models, the model utilizing the FT(3) embedding algorithm achieves an accuracy value of 93.85%. The combination of the FT(2) embedding algorithm with the BiLSTM architecture yields the highest accuracy value with 96.15%. Conversely, the model incorporating the FT(4) embedding algorithm with the BiLSTM architecture again demonstrates the lowest accuracy value of 67.20%. Furthermore, the FT(2)+BiLSTM model indicates the ability to detect positive classes at 92.80% and negative classes at 95.65%, respectively when considering sensitivity, and specificity metrics. The results presented in Table 9 demonstrate the most successful outcomes obtained after hyper-parameter tuning that is represented for Independent dataset as Table S8.

**Table 9:** Experiment results of FastText embedding model including various N-grams with deep learning algorithms for independent dataset. The best accuracy percentages are represented in bold letters.

**MODELS**	**ACC**	**SEN**	**SPE**	**MCC**	**AUC**
FT(2)+CNN	92.18	92.45	91.76	84.02	96.83
FT(3)+CNN	93.75	93.62	93.18	87.56	97.54
FT(4)+CNN	57.81	59.82	53.64	15.21	62.43
FT(2)+LSTM	87.50	88.24	86.36	73.86	91.57
FT(3)+LSTM	89.06	87.32	90.12	76.03	92.74
FT(4)+LSTM	62.33	56.34	59.28	16.07	58.89
FT(2)+BiLSTM	**96.15**	92.80	95.65	88.85	93.20
FT(3)+BiLSTM	93.85	92.87	94.63	87.41	96.85
FT(4)+BiLSTM	67.20	55.79	59.83	15.74	60.29

Drawing upon the insights derived from Table 9, it conspicuously emerges that the judicious selection of FastText n-gram embedding algorithms and deep learning architectural configurations exerts a profound influence on the overall model accuracy. The FT(3) embedding algorithm consistently exhibits remarkable prowess, consistently yielding superior accuracy metrics across both CNN and LSTM models. Remarkably, the FT(2) algorithm excels, particularly in tandem with the BiLSTM model, emerging as the optimal choice for achieving the highest classification performance. It is worth noting that this algorithm also demonstrates commendable performance, particularly within the CNN and LSTM models. In stark contrast, the FT(4) embedding algorithm persistently records the least favorable accuracy values across all architectural configurations. The amalgamation of insights from both Tables 8 and 9 underscores a noteworthy revelation that the strategic integration of the BiLSTM model engenders a substantial enhancement in the classification performance, particularly when harmonized with an optimally determined number of FastText n-grams for word embedding.

In Tables 10 and 11, the literature comparison is presented providing proposed models in the studies and accuracy scores. It is obviously observed that the proposed FT+BiLSTM framework exhibits remarkable experiment results with 92.50% of accuracy for ACPs250 dataset and 96.15% of accuracy for Independent dataset compared to the state-of-the-art studies. To demonstrate the computational efficiency of the proposed model, the performance of each model on two different dataset is presented in terms of training and inference time in Table 12. FastText has a unique feature as a word embedding model and takes subword information into account and goes beyond word roots. This provides better representations for rare words or specific terms, allowing the model to require less data and learn faster. FastText word embedding model can work with lower-dimensional vectors compared to other word embedding models. This results in reduced memory usage and faster processing because mathematical operations are faster with smaller dimensions. This allows the model to perform fewer unnecessary calculations and produce faster results. One-Hot Encoding requires high-dimensional vectors to create word representations. This increases memory usage and consumes more processing resources. When the BiLSTM model is combined with FastText, more compact and meaningful representations are used, allowing the model to produce faster results. FastText word embedding model is trained faster compared to other word embedding techniques. This enables the model to learn more quickly and deliver faster results.

**Table 10:** Comparison with literature studies for ACPs250 dataset. The best accuracy percentages are represented in bold letters.

**Study**	**Model**	**Accuracy**
Hajisharifi et al. (2014)	Hajisharifi	76.8
Akbar et al. (2017)	IACP	74.4
Wei et al. (2018)	ACPred-FL	88.4
Lawrence et al. (1997)	CNN	78.6
Lawrence et al. (1997)	DeepACP	82.9
Sun et al. (2022)	ACPNet	89.6
**Proposed method**	FT(3)+BiLSTM	**92.50**

**Table 11:** Comparison with literature studies for independent dataset. The best accuracy percentages are represented in bold letters.

**Study**	**Model**	**Accuracy**
Charoenkwan et al. (2021)	iACP-FSCM	82.50
Boopathi et al. (2019)	mACPpred	91.40
Liang et al. (2021)	ACPredStackL	92.40
Chen et al. (2016)	iACP	92.67
Li & Wang (2016)	Fm-Li	93.61
Akbar et al. (2022)	cACP-DeepGram	94.02
**Proposed method**	FT(2)+BiLSTM	**96.15**

**Table 12:** Computational performances of the best combinations on ACP250 and independent datasets. The best accuracy percentages are represented in bold letters.

**Models**	**Dataset**	**Training time**	**Inference time**
GL+BiLSTM	ACP250	6 m 2 s	8.24 s
GL+BiLSTM	Independent	6 m 11 s	8.62 s
OHE+BiLSTM	ACP250	7 m 40 s	12.76 s
OHE+BiLSTM	Independent	7 m 56 s	14.90 s
WC+BiLSTM	ACP250	5 m 45 s	7.13 s
WC+BiLSTM	Independent	5 m 57 s	7.32 s
FT(3)+BiLSTM	ACP250	**5 m 20 s**	**5**.**60 s**
FT(2)+BiLSTM	Independent	**5 m 18 s**	**5**.**21 s**

**Figure 9:**
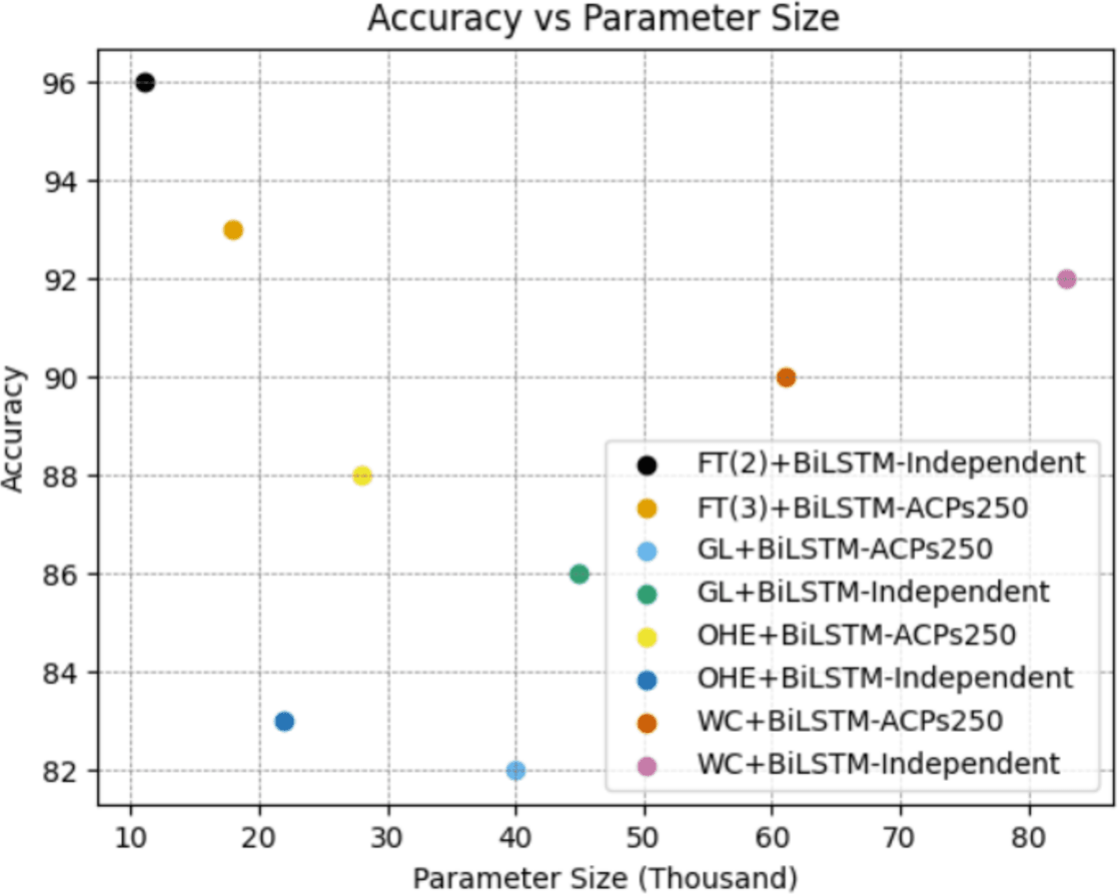
Evaluation of parameter size and classification performance for the best combinations on ACP250 and Independent datasets.

As clearly seen from the Fig. 9, the FastText+BiLSTM combination we recommended for both datasets has a low number of parameters, and this low complexity has a positive impact on its performance. A low number of parameters enables the model to generalize better and reduces the risk of overfitting. This supports the model in achieving successful results across a wider range of data. Furthermore, the model’s low complexity requires less memory and processor resources, making it a more efficient option for application and deployment. Therefore, the model we recommend strikes a notable balance with its low parameter count and high performance.

**Figure 10:**
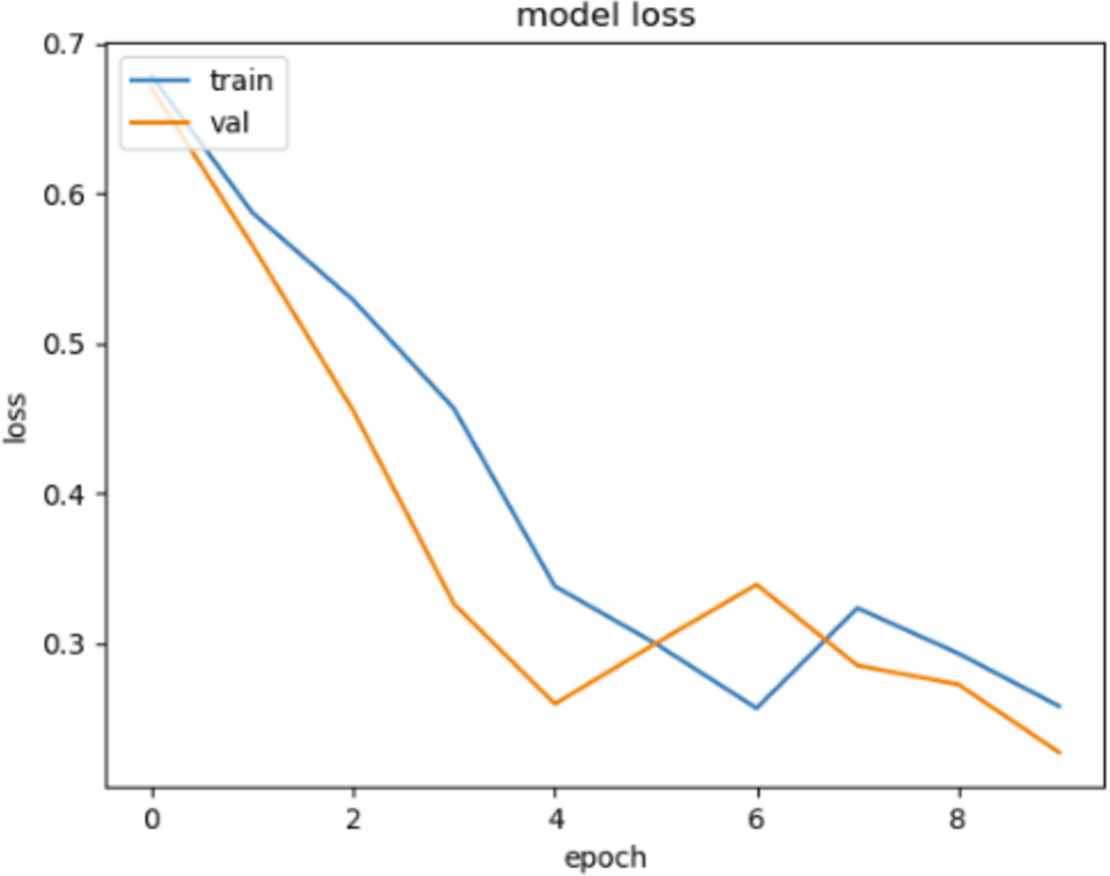
The loss graphic for proposed FT(3)+BiLSTM model of ACPs250 dataset.

**Figure 11:**
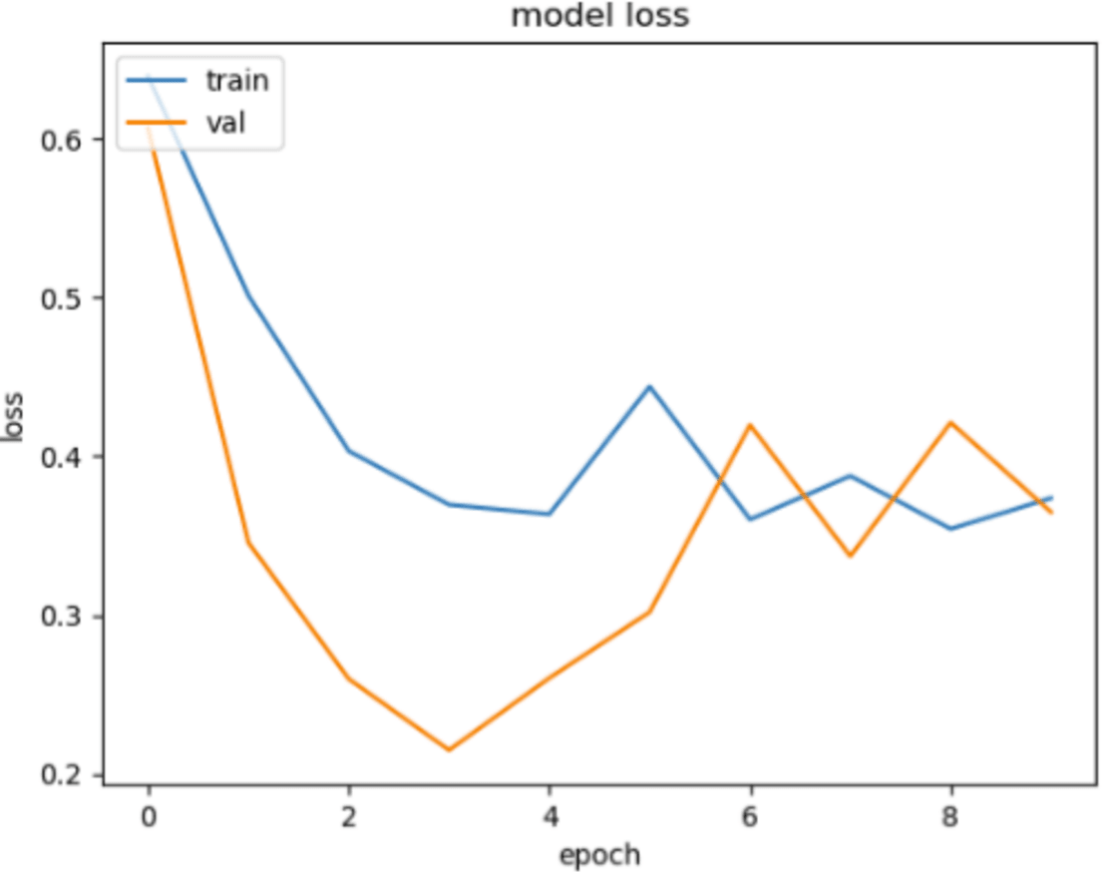
The loss graphic for proposed FT(2)+BiLSTM model of independent dataset.

In Figs. 10 and 11, loss movements according to epoch size of training and validation sets are presented for ACPs250 and Independent datasets. In Fig. 10, early-stopping criteria, drop-out, and regularization techniques are applied to prevent the over-fitting problem. Thence, the training process is ended at epoch size 4. This means the best classification result is ensured with 92.50% of accuracy at epoch size 4 by handling over-fitting problem. It is very clear from the graph that the training and validation losses decrease in parallel with the number of trials until the 4th epoch size for ACPs250 dataset. In Fig. 11, the application of early-stopping criteria, drop-out, and regularization techniques is observed to mitigate the issue of over-fitting, as in Fig. 10. Consequently, the training process culminates at an epoch size of 3 guaranteeing the attainment of the most favorable classification outcome with an impressive accuracy rate of 96.15%. Notably, this achievement is attributed to the successful management of the over-fitting problem. The graphical representation vividly portrays a synchronized decline in both training and validation losses as the number of iterations progresses, effectively demonstrating the favorable convergence of these metrics up until the 3rd epoch size for the Independent dataset.

The ROC curve serves as a metric for evaluating the performance of a classification model. The sharpness of the curve is associated with the model’s classification capability and error rate. A sharper ROC curve typically represents a well-performing model. In this case, the model may exhibit high accuracy, low error rate, and proficient classification ability. This implies that the model’s predictions provide a clearer distinction between the true classes. Conversely, a less sharp ROC curve may indicate lower classification performance for the model or a higher number of misclassifications in the confusion matrix. The sharpness of the ROC curve can depend on various factors, such as the features employed by the model, the training data, the complexity of the model, and the classification algorithm utilized. Optimizing or modifying these factors can influence the sharpness of the ROC curve. In conclusion, a sharper ROC curve signifies that the model demonstrates superior classification performance and provides a clearer differentiation between its predictions and the true classes.

**Figure 12:**
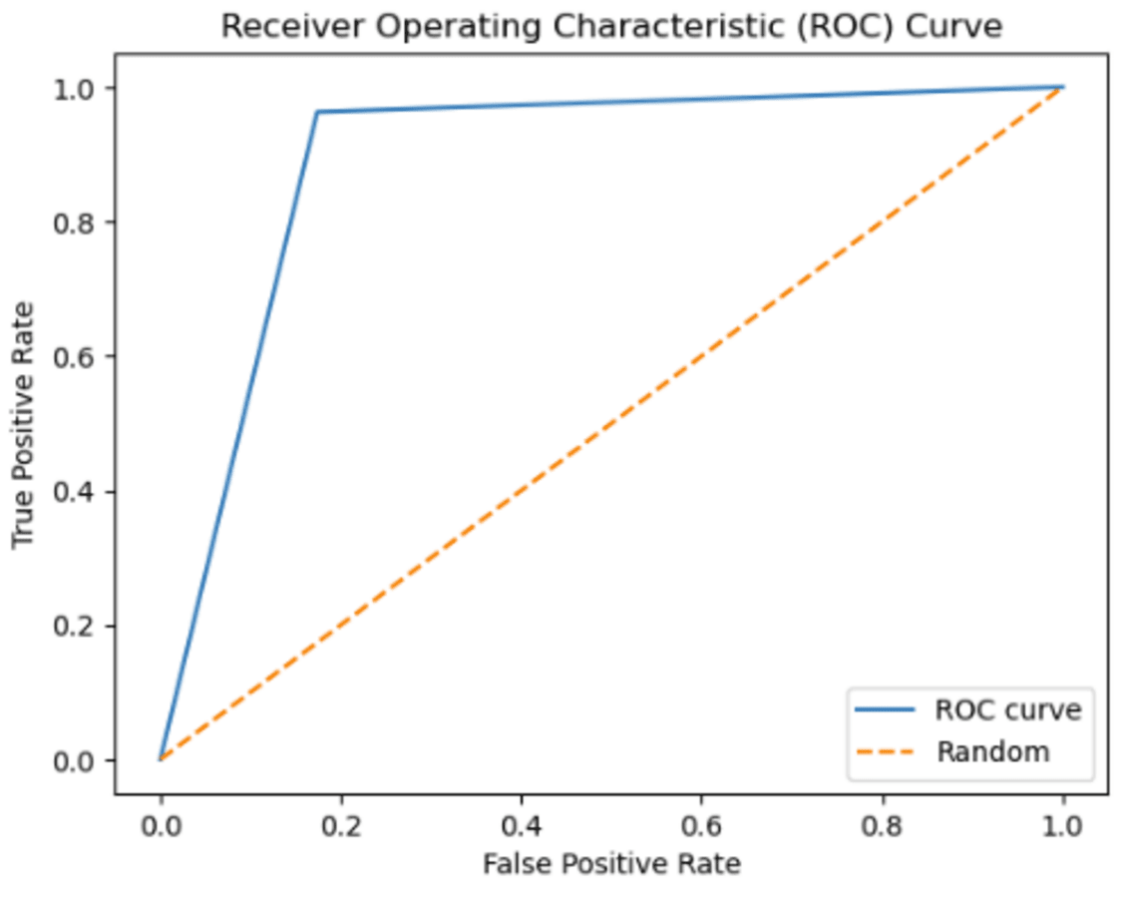
The ROC curve graphic for proposed FT(3)+BiLSTM model of ACPs250 dataset.

**Figure 13:**
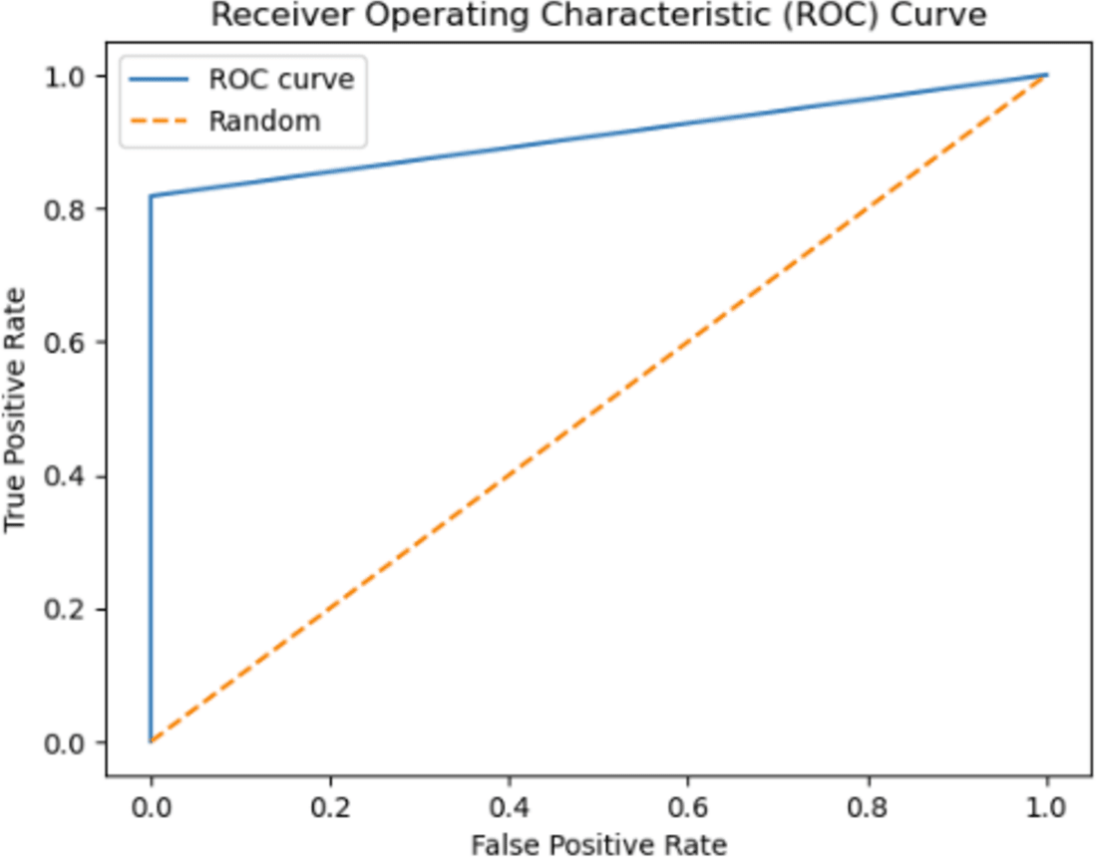
The ROC curve graphic for proposed FT(2)+BiLSTM model of independent dataset.

Instead of focusing solely on prediction rates, the researchers have employed AUC as a metric, illustrated in Figs. 12 and 13. The ROC curve serves as the primary tool for assessing the model’s stability and consistency. Without the ROC curve, there is no means to verify the model’s consistent performance across multiple points. It is possible that the model excels in one parameter while performing poorly in others. In this study, the highest AUC value of 89.45% was achieved using 3-gram descriptors, as depicted in Fig. 12, while 2-gram descriptors exhibited an AUC value of 93.20% in Fig. 13.

## Conclusions

This work introduces an efficient model for classifying anticancer peptides by combining word embedding techniques and deep learning models. The proposed framework utilizes Word2Vec, GloVe, FastText, and One-Hot-Encoding models to extract peptide sequences, which are then fed into CNN, LSTM, and BiLSTM architectures. Extensive experiments are conducted on well-known datasets in this field, namely ACPs250 and Independent, to evaluate the performance of the proposed model. Throughout the study, skip-gram and CBOW versions of Word2Vec model, GloVe, 2, 3, and 4 gram versions of FastText, and One-Hot-Encoding embedding algorithms are employed. Additionally, to enhance the reliability and performance of the models, a combination of techniques such as Dropout, pooling layer, and batch normalization are utilized. The experimental results demonstrate the effectiveness of the proposed approach, surpassing the success rate (89.6%) of state-of-the-art study (Sun et al., 2022) on the ACPs250 dataset and achieving a remarkable accuracy of 92.50%. Moreover, on the Independent dataset, the proposed model outperforms the leading study’s (Akbar et al., 2022) accuracy rate of 94.02% and achieves an impressive success rate of 96.15%.

There are many factors that affect the classification accuracy of the model. First, the choice of feature representation is crucial in ACP task. FastText embeddings provide a rich source of semantic information. When applied to amino acid sequences in anticancer peptide classification, FastText captures the semantic relationships between different amino acids and their role in peptide function. This semantic understanding is essential for identifying anticancer peptides with distinct characteristics. Second, the selection of convenient model is another significant stage for classifying anticancer peptides. BiLSTM is highly effective at capturing sequential dependencies and context within peptide sequences. It considers both the past and future amino acids when making predictions, which is crucial for recognizing specific sequence motifs and patterns unique to anticancer peptides. Third is the generalization capability of the combination of word embedding model and deep learning algorithm. The combination of FastText and BiLSTM enables the model to generalize from limited data. FastText embeddings are pre-trained on extensive text corpora, which provides the model with general language knowledge. This generalization ability is particularly valuable when working with small anticancer peptide datasets, as it helps the model identify common linguistic patterns. Furthermore, careful tuning of hyperparameters, such as the number of LSTM layers, dropout rates, and learning rates, is crucial for maximizing the model’s accuracy. On the other hand, there are challenges of the current model in terms of computational intensity, data size dependency, and over-fitting. BiLSTM models can be computationally intensive, especially if they have a large number of parameters or deep layers. Training and deploying such models may require substantial computational resources, which can be a limitation in resource-constrained environments. To cope with this issue, experiments are carried out on Google-Colab environment using free GPUs. Deep learning models like BiLSTM tend to require substantial amounts of training data to perform well. If the dataset is relatively small, the model might struggle to generalize effectively, potentially leading to over-fitting. Over-fitting can be a concern, particularly when dealing with limited data. If the model overfits, it may perform exceptionally well on the training data but fail to generalize to unseen peptides. To tackle with this problem, extensive experiments are carried out by performing hyperparameter tuning using such as early-stopping criteria, drop-out, and regularization techniques.

The findings provide valuable insights into the strengths, limitations, and potential areas of improvement for deep learning-based approaches in ACP classification, informing future research in this field. The significance of this research lies in the development of efficient computational tools for accurate ACP classification, which can facilitate the discovery of novel anticancer therapeutics. The comparative analysis of deep learning models contributes to the existing knowledge in the field and shed light on their applicability and suitability for ACP classification. Ultimately, this research aims to enhance our understanding of ACPs and pave the way for the design of more effective and targeted anticancer treatments.

## Supplemental Information

10.7717/peerj-cs.1831/supp-1 Supplemental Information 1 Sample of hyper-parameter tuning results of GL+BiLSTM for ACP250 Dataset

10.7717/peerj-cs.1831/supp-2 Supplemental Information 2 Sample of hyper-parameter tuning results of OHE+BiLSTM for ACP250 Dataset

10.7717/peerj-cs.1831/supp-3 Supplemental Information 3 Sample of hyper-parameter tuning results of GL+BiLSTM model for Independent Dataset

10.7717/peerj-cs.1831/supp-4 Supplemental Information 4 Sample of hyper-parameter tuning results of OHE+BiLSTM model for independent dataset

10.7717/peerj-cs.1831/supp-5 Supplemental Information 5 Sample of hyper-parameter tuning results of WC+BiLSTM model for ACP250 dataset

10.7717/peerj-cs.1831/supp-6 Supplemental Information 6 Sample of hyper-parameter tuning results of WC+BiLSTM model for independent dataset

10.7717/peerj-cs.1831/supp-7 Supplemental Information 7 Sample of hyper-parameter tuning results of FT(3)+BiLSTM model for ACP250 dataset

10.7717/peerj-cs.1831/supp-8 Supplemental Information 8 Sample of hyper-parameter tuning results of FT(2)+BiLSTM model for independent dataset

## Additional Information and Declarations

### Data Availability

The following information was supplied regarding data availability: The datasets are available in the Supplemental Files and at GitHub: - ACPs250 dataset: https://github.com/abcair/ACPNet/tree/main/data
- Independent dataset: https://github.com/shahidakbarcs/cACP-DeepGram/blob/main/Datasets/Independent%20Dataset/Independent_dataset.txt
The Python source code for the study are available in Zenodo: Karakaya, O. (2024). Python files where datasets and studies are performed. Zenodo. https://doi.org/10.5281/zenodo.10521827.

## References

[ref-1] Adeel A, Gogate M, Hussain A (2020). Contextual deep learning-based audio-visual switching for speech enhancement in real-world environments. Information Fusion.

[ref-2] Ahmed S, Muhammod R, Khan ZH, Adilina S, Sharma A, Shatabda S, Dehzangi A (2021). ACP-MHCNN: an accurate multi-headed deep-convolutional neural network to predict anticancer peptides. Scientific Reports.

[ref-3] Akbar S, Hayat M, Iqbal M, Jan MA (2017). iACP-GAEnsC: evolutionary genetic algorithm based ensemble classification of anticancer peptides by utilizing hybrid feature space. Artificial Intelligence in Medicine.

[ref-4] Akbar S, Hayat M, Tahir M, Khan S, Alarfaj FK (2022). cACP-DeepGram: classification of anticancer peptides via deep neural network and skip-gram-based word embedding model. Artificial Intelligence in Medicine.

[ref-5] Al-Dulaimi K, Chandran V, Nguyen K, Banks J, Tomeo-Reyes I (2019). Benchmarking HEP-2 specimen cells classification using linear discriminant analysis on higher order spectra features of cell shape. Pattern Recognition Letters.

[ref-6] Alom MZ, Taha TM, Yakopcic C, Westberg S, Sidike P, Nasrin MS, Asari VK (2019). A state-of-the-art survey on deep learning theory and architectures. Electronics.

[ref-7] Alsanea M, Dukyil AS, Afnan R, Riaz B, Alebeisat F, Islam M, Habib S (2022). To assist oncologists: an efficient machine learning-based approach for anti-cancer peptides classification. Sensors.

[ref-8] Amrit C, Paauw T, Aly R, Lavric M (2017). Identifying child abuse through text mining and machine learning. Expert Systems with Applications.

[ref-9] Aziz AZB, Hasan MAM, Ahmad S, Al Mamun M, Shin J, Hossain MR (2022). iACP-MultiCNN: multi-channel CNN based anticancer peptides identification. Analytical Biochemistry.

[ref-10] Bojanowski P, Grave E, Joulin A, Mikolov T (2017). Enriching word vectors with subword information. Transactions of the Association for Computational Linguistics.

[ref-11] Boopathi V, Subramaniyam S, Malik A, Lee G, Manavalan B, Yang D-C (2019). mACPpred: a support vector machine-based meta-predictor for identification of anticancer peptides. International Journal of Molecular Sciences.

[ref-12] Charoenkwan P, Chiangjong W, Lee VS, Nantasenamat C, Hasan M, Shoombuatong W (2021). Improved prediction and characterization of anticancer activities of peptides using a novel flexible scoring card method. Scientific Reports.

[ref-13] Chen W, Ding H, Feng P, Lin H, Chou K-C (2016). iACP: a sequence-based tool for identifying anticancer peptides. Oncotarget.

[ref-14] Crawford M, Khoshgoftaar TM, Prusa JD, Richter AN, Al Najada H (2015). Survey of review spam detection using machine learning techniques. Journal of Big Data.

[ref-15] Feng G, Yao H, Li C, Liu R, Huang R, Fan X, Ge R, Miao Q (2022). ME-ACP: multi-view neural networks with ensemble model for identification of anticancer peptides. Computers in Biology and Medicine.

[ref-16] Ghoshal S, Rigney G, Cheng D, Brumit R, Gee M, Hodin R, Lillemoe K, Levine W, Succi M (2022). Institutional surgical response and associated volume trends throughout the COVID-19 pandemic and postvaccination recovery period. JAMA Network Open.

[ref-17] Ghulam A, Ali F, Sikander R, Ahmad A, Ahmed A, Patil S (2022). ACP-2DCNN: deep learning-based model for improving prediction of anticancer peptides using two-dimensional convolutional neural network. Chemometrics and Intelligent Laboratory Systems.

[ref-18] Graves A, Jaitly N, Mohamed A (2013). Speech recognition with deep recurrent neural networks.

[ref-19] Gregorc V, Braud F, Pas T, Scalamogna R, Citterio G, Milani A, Boselli S, Catania C, Danodoni G, Rossoni G, Ghio D, Spitaleri G, Ammannati C, Colombi S, Caligaris-Cappio F, Lambiase A, Bordignon C (2011). Phase I study of NGR-hTNF, a selective vascular targeting agent, in combination with cisplatin in refractory solid tumors. Clinical Cancer Research.

[ref-20] Hajisharifi Z, Piryaiee M, Beigi MM, Behbahani M, Mohabatkar H (2014). Predicting anticancer peptides with Chou’s Pseudo amino acid composition and investigating their mutagenicity via ames test. Journal of Theoretical Biology.

[ref-21] Hochreiter S, Schmidhuber J (1997). Long short-term memory. Neural Computation.

[ref-22] Holohan C, Van Schaeybroeck S, Longley DB, Johnston PG (2013). Cancer drug resistance: an evolving paradigm. Nature Reviews Cancer.

[ref-23] Hossain E, Khan I, Un-Noor F, Sikander SS, Sunny MSH (2019). Application of big data and machine learning in smart grid, and associated security concerns: a review. IEEE Access.

[ref-24] Khalili P (2006). A Non-RGD-Based Integrin Binding Peptide (ATN-161) blocks breast cancer growth and metastasis in vivo. Molecular Cancer Therapeutics.

[ref-25] Koppe G, Meyer-Lindenberg A, Durstewitz D (2021). Deep learning for small and big data in psychiatry. Neuropsychopharmacology.

[ref-26] Lawrence S, Giles CL, Tsoi AC, Back AD (1997). Face recognition: a convolutional neural-network approach. IEEE Transactions on Neural Networks.

[ref-27] LeCun Y, Bottou L, Bengio Y, Haffner P (1998). Gradient-based learning applied to document recognition. Proceedings of the IEEE.

[ref-28] Li F-M, Wang X-Q (2016). Identifying anticancer peptides by using improved hybrid compositions. Scientific Reports.

[ref-29] Liang X, Li F, Chen J, Li J, Wu H, Li S, Song J, Liu Q (2021). Large-scale comparative review and assessment of computational methods for anti-cancer peptide identification. Briefings in Bioinformatics.

[ref-30] Liu J, Li M, Chen X (2022). AntiMF: a deep learning framework for predicting anticancer peptides based on multi-view feature extraction. Methods.

[ref-31] Liu W, Wang Z, Liu X, Zeng N., Liu Y, Alsaadi FE (2017). A survey of deep neural network architectures and their applications. Neurocomputing.

[ref-32] Maliepaard M, Scheffer GL, Faneyte IF, Gastelen M, Pijnenborg A, Schinkel A, Vijver M, Scheper R, Schellens J (2001). Subcellular localization and distribution of the breast cancer resistance protein transporter in normal human tissues. Cancer Research.

[ref-33] Matthews HK, Bertoli C, de Bruin RAM (2022). Cell cycle control in cancer. Nature Reviews Molecular Cell Biology.

[ref-34] Mikolov T, Chen K, Corrado G, Dean J (2013). Efficient estimation of word representations in vector space.

[ref-35] Park HW, Pitti T, Madhavan T, Jeon YJ, Manavalan B (2022). MLACP 2.0: an updated machine learning tool for anticancer peptide prediction. Computational and Structural Biotechnology Journal.

[ref-36] Pennington J, Socher R, Manning CD (2014). Glove: global vectors for word representation.

[ref-37] Potok TE, Schuman C, Young S, Patton R, Spedalieri F, Liu J, Chakma G (2018). A study of complex deep learning networks on high-performance, neuromorphic, and quantum computers. ACM Journal on Emerging Technologies in Computing Systems (JETC).

[ref-38] Pouyanfar S, Sadiq S, Yan Y, Tian H, Tao Y, Reyes MP, Iyengar S (2018). A survey on deep learning: algorithms, techniques, and applications. ACM Computing Surveys (CSUR).

[ref-39] Rozenwald MB, Galitsyna AA, Sapunov GV, Khrameeva EE, Gelfand MS (2020). A machine learning framework for the prediction of chromatin folding in drosophila using epigenetic features. PeerJ Computer Science.

[ref-40] Sun M, Yang S, Hu X, Zhou Y (2022). ACPNet: a deep learning network to identify anticancer peptides by hybrid sequence information. Molecules.

[ref-41] Thundimadathil J (2012). Cancer treatment using peptides: current therapies and future prospects. Journal of Amino Acids.

[ref-42] Tian H, Chen SC, Shyu ML (2020). Evolutionary programming based deep learning feature selection and network construction for visual data classification. Information Systems Frontiers.

[ref-43] Tyagi A, Tuknait A, Anand P, Gupta S, Sharma M, Mathur D, Joshi A, Singh S, Gautam A, Raghava G (2015). CancerPPD: a database of anticancer peptides and proteins. Nucleic Acids Research.

[ref-44] Wei L, Zhou C, Chen H, Song J, Su R (2018). ACPred-FL: a sequence-based predictor using effective feature representation to improve the prediction of anti-cancer peptides. Bioinformatics.

[ref-45] Wu C, Gao R, Zhang Y, De Marinis Y (2019). PTPD: predicting therapeutic peptides by deep learning and Word2Vec. BMC Bioinformatics.

[ref-46] Xin Y, Kong L, Liu Z, Chen Y, Li Y, Zhu H, Wang C (2018). Machine learning and deep learning methods for cybersecurity. IEEE Access.

[ref-47] Yabroff KR, Wu XC, Negoita S, Stevens J, Coyle L, Zhao J, Mumphrey B, Jemal A, Ward K (2022). Association of the COVID-19 pandemic with patterns of statewide cancer services. Journal of the National Cancer Institute.

[ref-48] Young T, Hazarika D, Poria S, Cambria E (2018). Recent trends in deep learning based natural language processing. IEEE Computational Intelligence Magazine.

[ref-49] Yu L, Jing R, Liu F, Luo J, Li Y (2020). DeepACP: a novel computational approach for accurate identification of anticancer peptides by deep learning algorithm. Molecular Therapy-Nucleic Acids.

