# Supplementary material for: An efficient consolidation of word embedding and deep learning techniques for classifying anticancer peptides: FastText+BiLSTM
Source: PeerJ Comput Sci. 2024 Feb 20;10:e1831. doi: 10.7717/peerj-cs.1831 (PMC10909209; doi:10.7717/peerj-cs.1831)
Supplement: Supplemental Information 8 [file peerj-cs-10-1831-s008.docx]

| Trials | Epoch Size | Drop Out | Batch Size | Learning Rate | Activation Function | Accuracy |
| --- | --- | --- | --- | --- | --- | --- |
| 1 | 15 | 0.5 | 128 | 0.001 | softmax | 48.43 |
| 2 | 15 | 0.5 | 128 | 0.001 | softmax | 48.43 |
| 3 | 15 | 0.4 | 32 | 0.0001 | sigmoid | 51.56 |
| 4 | 10 | 0.3 | 32 | 0.001 | sigmoid | 85.93 |
| 5 | 5 | 0.2 | 64 | 0.001 | sigmoid | 89.06 |
| 6 | 5 | 0.2 | 64 | 0.01 | sigmoid | **96.15** |
